# Activation of Gαq sequesters specific transcripts into Ago2 particles

**DOI:** 10.1038/s41598-022-12737-w

**Published:** 2022-05-24

**Authors:** Lela Jackson, Madison Rennie, Alison Poussaint, Suzanne Scarlata

**Affiliations:** Department of Chemistry and Biochemistry, Worcester Polytechnic Institute, 100 Institute Rd, Worcester, MA 01609 USA

**Keywords:** Biochemistry, Biophysics, Physiology

## Abstract

The Gαq/phospholipase Cβ1 (PLCβ1) signaling system mediates calcium responses from hormones and neurotransmitters. While PLCβ1 functions on the plasma membrane, there is an atypical cytosolic population that binds Argonaute 2 (Ago2) and other proteins associated with stress granules preventing their aggregation. Activation of Gαq relocalizes cytosolic PLCβ1 to the membrane, releasing bound proteins, promoting the formation of stress granules. Here, we have characterized Ago2 stress granules associated with Gαq activation in differentiated PC12 cells, which have a robust Gαq/PLCβ1 signaling system. Characterization of Ago2-associated stress granules shows shifts in protein composition when cells are stimulated with a Gαq agonist, or subjected to heat shock or osmotic stress, consistent with the idea that different stresses result in unique stress granules. Purified Ago2 stress granules from control cells do not contain RNA, while those from heat shock contain many different mRNAs and miRs. Surprisingly, Ago2 particles from cells where Gαq was stimulated show only two transcripts, chromogranin B, which is involved in secretory function, and ATP synthase 5f1b, which is required for ATP synthesis. RT-PCR, western blotting and other studies support the idea that Gαq-activation protects these transcripts. Taken together, these studies show a novel pathway where Gαq/PLCβ regulates the translation of specific proteins.

## Introduction

Binding of extracellular ligands such as acetylcholine, serotonin and histamine, to their specific G protein coupled receptor will activate Gαq, one of the four major G proteins pathways^1^. Gαq, in turn, activates phospholipase Cβ, which catalyzes the hydrolysis of the signaling lipid phosphoinositide 4,5 bisphosphate leading to an increase in intracellular calcium^2^. Along with this important membrane function, PLCβ1 has been shown to have a cytosolic population that binds to the Promoter of RNA-induced silencing, C3PO, as well as several proteins involved in stress granules formation^3,4^. Stress granules are halted ribosomal complexes that protect mRNAs under stress conditions such as arsenite treatment, heat/cold shock and osmotic stress^5–7^. Proteins that bind PLCβ1 include eFI5A, polyadenylate binding protein (PABPC1) and Ago2. Ago2 is additionally the main nuclease component of the RNA-induced silencing complex (RISC)^8^ that degrades mRNA with the help of C3PO. When Ago2-bound mRNA pairs perfectly with a bound miR, Ago2 transitions to an active conformation to hydrolyze the mRNA. However, if pairing is imperfect, it will form a stalled complex^9–11^ resulting in stress granules.

Our recent study showed that reducing the cytosolic PLCβ1 population increases the number and size of particles containing Ago2 as well as the stress granule markers PABPC1 and G3BP1^4,12^. Binding between PLCβ1 and stress granule proteins helps keep them disperse, while activation of Gαq promotes relocalization of cytosolic PLCβ1 to the plasma membrane, promoting release of bound proteins and the formation of stress granules. This mechanism suggests that Gαq may be connected to protein translation through cytosolic PLCβ1.

Here, we have characterized the composition of Ago2 stress granules formed in response to Gαq activation in differentiated PC12 cells, and compared these to traditional stress responses. PC12 cells have a large endogenous expression of Gαq and PLCβ1, and although not neuronal in origin, when treated with nerve growth factor, the cells adopt neuronal morphology and secrete particles mimicking synaptic vesicles^13,14^. We find that Gαq activation produces stress granules that have a distinct protein composition as compared to other stresses. Also, unlike heat shock that contain different mRNA and miRs, Gαq stress granules contain only two major mRNA transcripts. Our studies show a surprising connection between physiological Gαq activation and protein translation.

## Results

### Activation of Gαq promotes stress responses in differentiated cells

**Figure 1:**
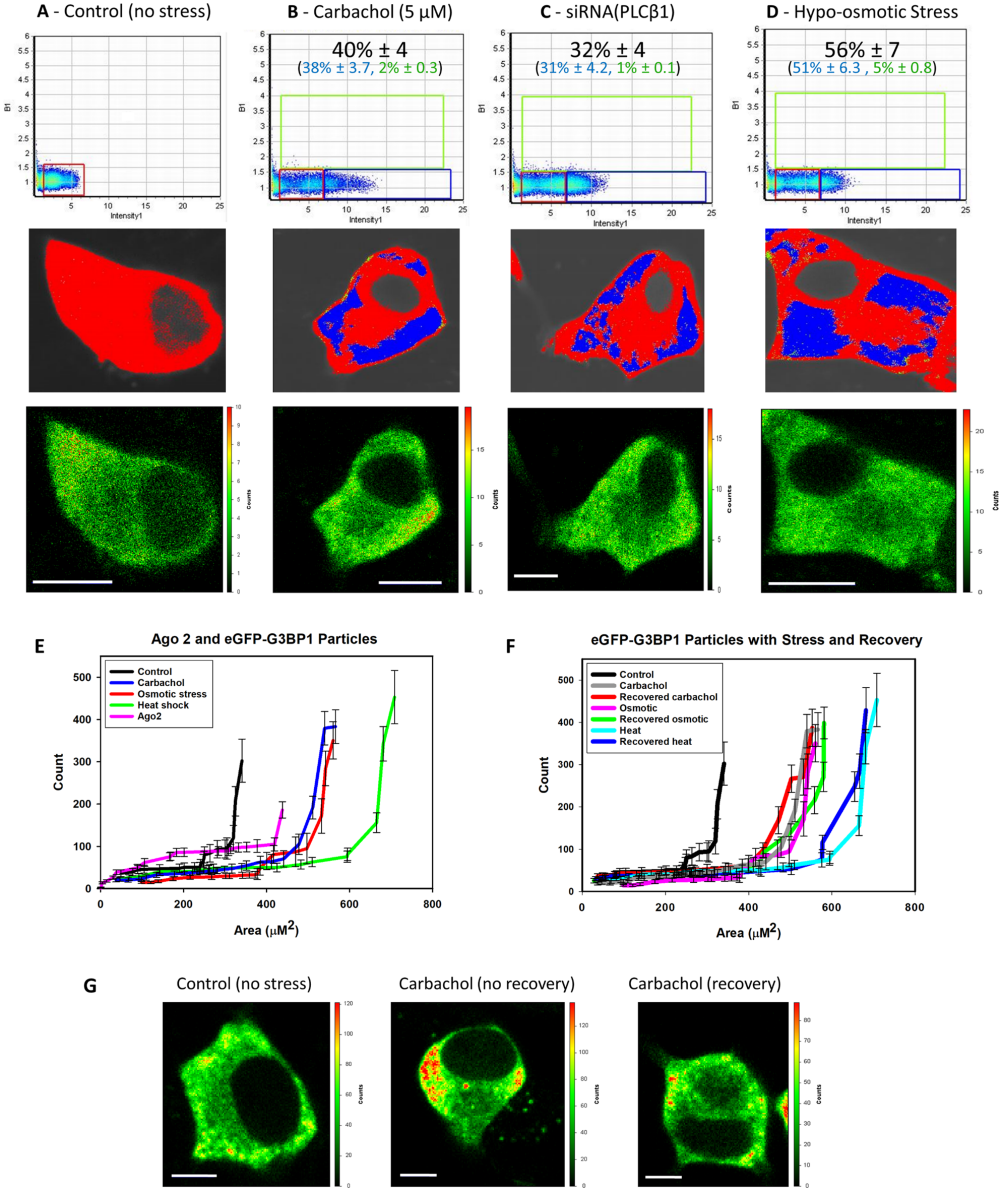
Aggregation of G3BP1 in differentiated PC12 cells. Cells transfected with eGFP-G3BP1 were at (**A)** control conditions, n = 12; (**B**) activated Gαq by exposure to carbachol for 10 min, n = 30; (**C**) treated with siRNA(PLCβ1), n = 28; (**D)** subjected to hypo-osmotic stress (150 mOsm) for 5 min, n = 14. The graphs in the top rows display N&B values for each pixel where the y-axis corresponds to the brightness of the particle and the x-axis shows particle intensity. The pixels contained in the red boxes are the values found for free, non-aggregated eGFP. Points outside the red boxes shown in blue correspond to a high number of condensed monomeric protein, while points shown in green correspond to higher-order species of G3BP1 where the percent of pixels in these categories are given. Images in the middle rows show the pixels corresponding to free (red) and aggregated eGFP-G3BP1 (green and blue). Images in the bottom row are the corresponding fluorescence microscopy images. Total percent protein aggregation for each condition is reported in red text, while the portion of total aggregation derived from the green and blue boxes is reported in green and blue text, respectively. We note that a significant percent of cells in the carbachol (66%) and siRNA(PLCβ1) (54%) samples showed low (~ 2%) aggregation and only the percentages in the higher groups are shown. Scale bars = 10 μm. (**E**) Cumulative values of the intensities (count) and size of eGFP-Ago2 or eGFP-G3BP1 particles in live cells under control conditions as determined by confocal imaging where the count values for Ago2 are reduced by tenfold for scaling purposes. Also shown are eGFP-G3BP1 particle size and number in live cells immediately after carbachol treatment, hypo-osmotic stress or heat shock. For all conditions, n = 11 for eGFP-G3BP1 and n = 12 for Ago2 where SD is shown. **(F)** eGFP-G3BP1 particle data shown in **(E)** along with data taken for cells that were treated to a stress condition and then allowed to recover at 37 °C for 30 min where n = 11 and SE is shown. **(G)** sample images of PC12 cells expressing eGFP-Ago2 with stimulation and recovery. Scale bars = 10 µm.

Previous studies followed the formation of stress granules associated with Ago2 and PABPC1 by Gαq activation in undifferentiated PC12 cells as well as two other cell lines^4^, and here we extended these studies to differentiated PC12 cells. We first followed the assembly of the stress granule marker, eGFP-G3BP1, whose dimerization initiates stress granule formation^15^. These studies used time-lapse fluorescence imaging and assessed the number of fluorophores associated with a diffusing particle called Number and Brightness (N&B)^16^ analysis. We find that the amount of eGFP-G3BP1 fluorescence intensity associated with diffusing particles increases with the loss of cytosolic PLCβ1 either by down-regulation, by relocalization to the membrane upon Gαq activation, or by osmotic stress which also reduces cytosolic PLCβ1 levels^4^ (Fig. 1A–D). Although much of the G3BP1 aggregation that we see in N&B measurements is localized monomeric protein (denoted in blue text), there is also a fraction of detected oligomeric protein and that portion is reported in green text. It is important to note that we are inducing aggregation by different stresses where G3BP1 dimerization might play a lesser role. Additionally, aggregation of the fluorophores raises the possibility of photophysical mechanisms that reduce fluorescence such as homotransfer, which is highly likely given the close proximity of the fluorophores and/or self-quenching^17^.

Additionally, we followed the formation of larger eGFP-G3BP1 aggregates in live cells when subjected to Gαq stimulation and compared these data to heat shock or osmotic stress by measuring their size and number by confocal imaging using a 100 × objective (Fig. 1E). As expected, we find a significant increase in the cumulative number and size of particles relative to control for all three stress conditions, consistent with stress granule formation^15^. The trends seen for eGFP-G3BP1 mirror previous studies^4^ of Ago2 under stress conditions. Removing the stress and viewing the cells 30 min after recovery show little disassembly of the stress granules (Fig. 1F,G). We note that G3BP1 particles formed under all conditions were stable for at least 30 min.

### Characterization of proteins associated with Ago2 under stress conditions

We characterized the proteins associated with Ago2 stress granules. These studies were carried out by pulling down Ago2 with a monoclonal antibody from the cytosolic fraction of cells subjected to Gαq stimulation, hypo-osmotic stress or heat shock. The proteins contained in these Ago2 complexes were then identified by mass spectrometry. We found that Ago2 complexes isolated from cells under control conditions showed the highest number of bound proteins (~ 125) accounting for more than ~ 95% of the total protein (SI Table 1). However, cells subjected to Gαq stimulation, heat shock and osmotic stress conditions had far less different types of proteins bound to Ago2 (SI Table 2–4).

**Figure 2:**
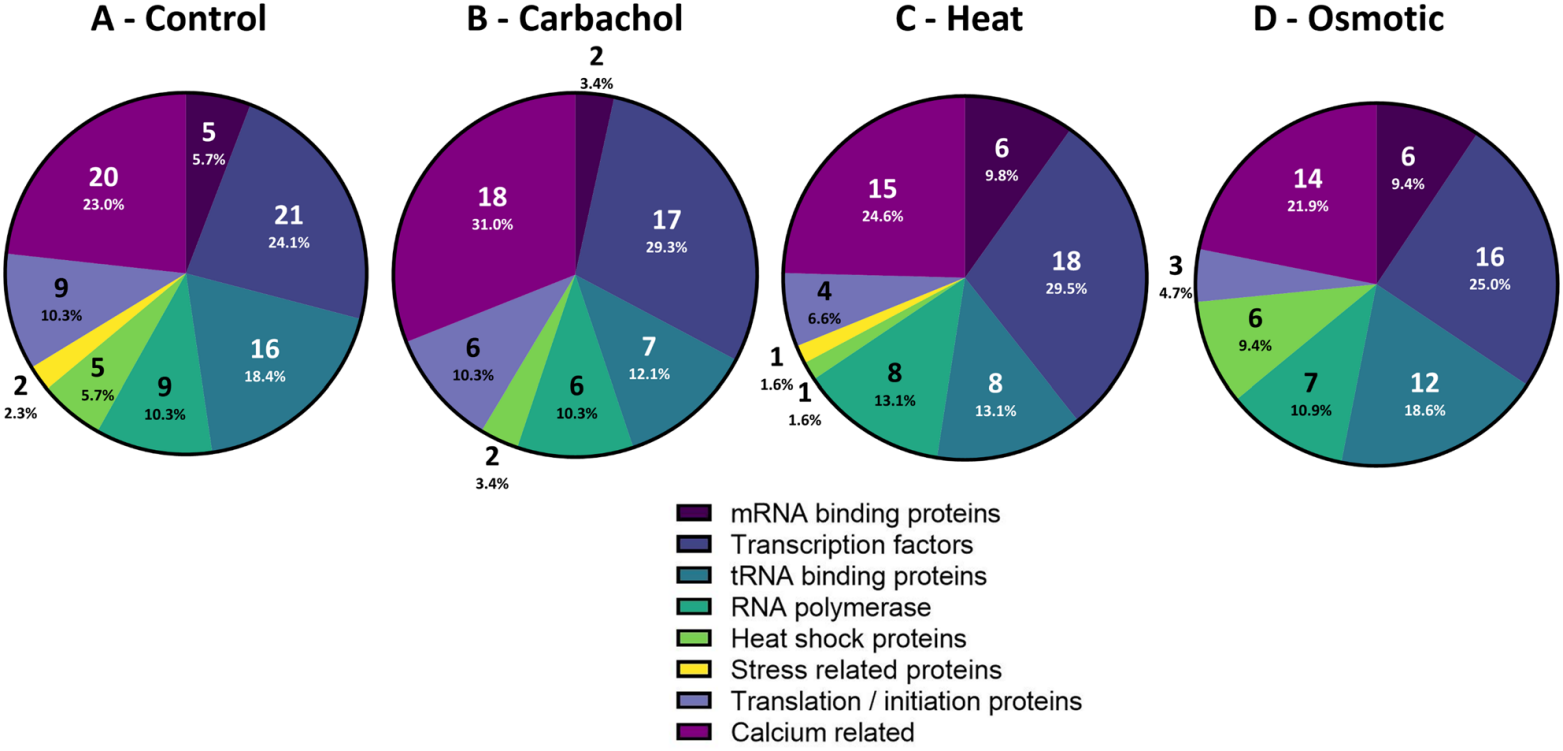
Distribution of Ago2 stress granule proteins under different stress. PC12 cells over expressing eGFP-Ago2 were exposed to **(A**) no stress, (**B**) carbachol activation of Gαq (10 min, 5 µM), (**C**) heat shock (1 h, 42 °C), or (**D**) hypo-osmotic stress (150 mOsm, 5 min). Ago2 particles were pulled down for cytosolic fractions using a monoclonal GFP antibody. Purified stress granules were then subject to mass spectrometry analysis and results were filtered by the following categories: mRNA binding proteins, transcription factors, tRNA binding proteins, RNA polymerases, heat shock proteins, stress related proteins, and translation/initiation proteins, and calcium-sensitive proteins where the values in the segment corresponds to the number of the total amount of Ago2-bound proteins.

We grouped the Ago2 bound proteins into functional categories: mRNA associated, transcription factors, tRNA associated, RNA polymerase, stress proteins, heat shock and eukaryotic initiation and translation, and proteins sensitive to changes in intracellular calcium (Fig. 2A–D) and we note that several cytoskeletal proteins appear. As expected, different conditions had different protein distributions relative to control. Proteins bound to Ago2 in cells subjected to heat shock and Gαq stimulation show an increased percentage of transcription factors, which is consistent with a halting of fundamental cellular events, whereas samples from cells subjected to osmotic stress show conserved levels of proteins associated with tRNA, which is consistent with shifts in protein production to restore basal osmolality. Comparison of the most abundant proteins associated with Ago2 under each condition shows transcriptional enhancer factor TEF-5 to be the major binding partner followed by cytoskeletal and structural proteins (i.e. actin, desmoplakin, titin, dynein, nesprin), whose amounts shift slightly with each condition. Focusing on the ten most abundant proteins, Ago2 complexes formed under Gαq stimulation showed only one unique protein, polyubiquitin-B, while both Ago2 complexes under Gαq stimulation and osmotic stress contain Phe tRNA ligase, which is absent in control and heat shock samples.

### Gαq activation is associated with Ago2 particles that contain specific transcripts

We sequenced the RNAs contained in Ago2 particles characterized above (SI Table 5–6). We could not detect RNA in Ago2 complexes pulled-down from control cells consistent with the absence of stable stress granules under basal conditions. In contrast, Ago2 particles from cells stimulated with Gαq or subjected to heat shock contained measurable amounts of RNAs, and these were isolated and sequenced. We found these two conditions yielded were very different results; in the Gαq-stimulated samples, only two transcripts of the ~ 50 most abundant RNAs were were seen, *chromogranin A/B* (*Chga/b*) and *ATPsynthase 5f1b* (*ATP5f1b*). In contrast heat shock samples contained an assortment of different mRNAs and miRs. These results suggest that Ago2 particles formed in response to Gαq sequester specific transcripts, while those formed from heat are non-specific.

We extended the above studies by measuring changes in the total cellular levels by RT-PCR under different conditions. Levels of *Chgb* and *ATP5f1b* with carbachol stimulation were measured. Because carbachol stimulates Gαi as well as Gαq, we treated cells with pertussis toxin to inactivate Gαi signals and did not see changes in *Chgb* or *ATP5f1b* levels. We additionally carried out other controls including stimulation of Gαi rather than Gαq by isoproterenol, and measurements of *tau* mRNA since this transcript is not found in Ago2 complexes in cells subjected to Gαq stimulation or heat shock, although protein levels of tau are influenced by the PLCβ1/Gαq pathway by a mechanism independent of stress granules^18^.

**Figure 3:**
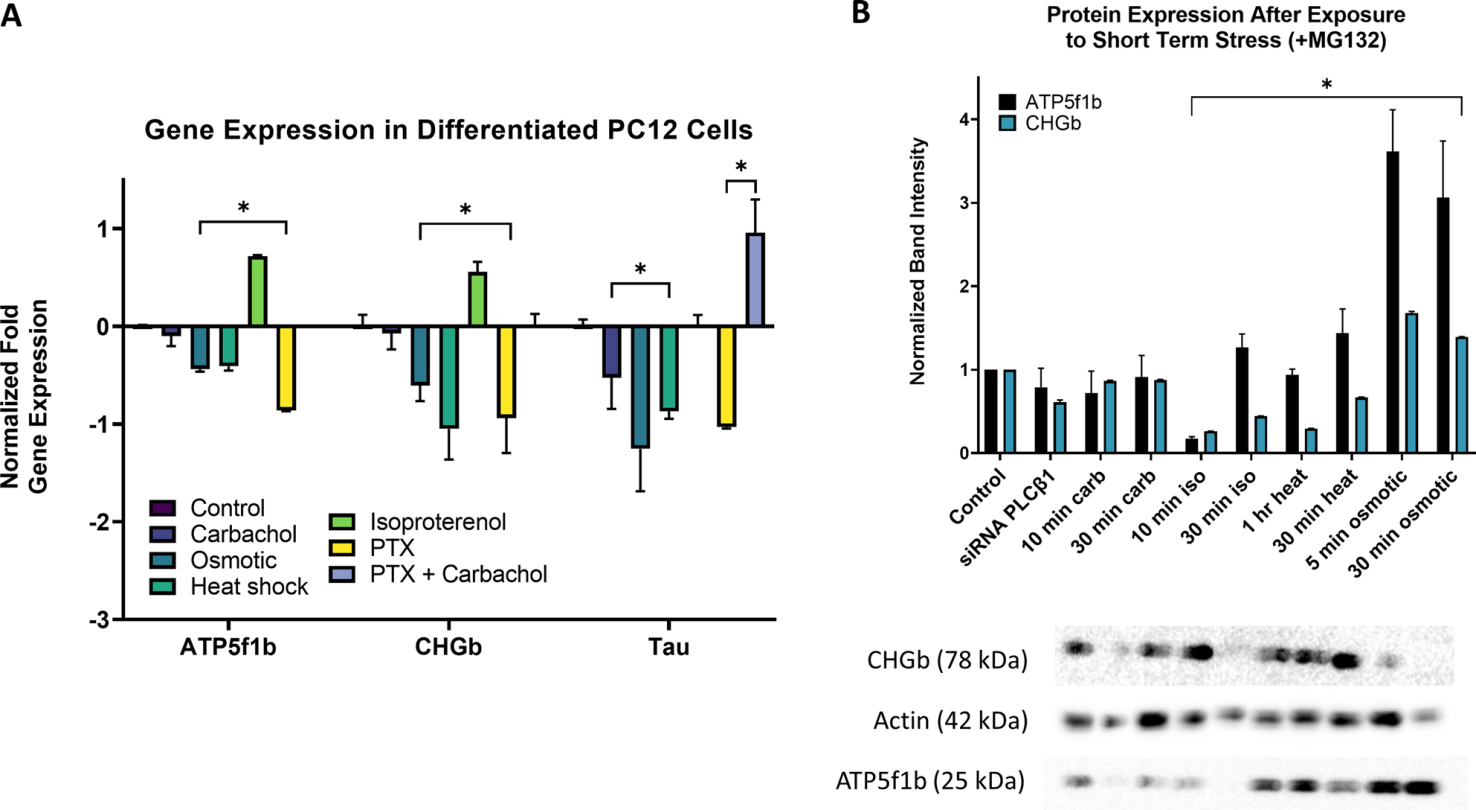
Gene expression and protein levels of ATP5f1b and CHGb in cells under stress. (**A**) RT-PCR results showing the relative levels of gene expression in PC12 cells of *ATP5f1b, Chgb* and *tau* (as a negative control) in differentiated PC12 cells exposed to different stress conditions (carbachol stimulation, hypo-osmotic stress, heat shock, isoproterenol as a Gαi stimulant and pertussis toxin (PTX) to eliminate effects of Gαi), where * denotes p ≤ 0.001 and n = 3–9 and SE is shown. (**B**) PC12 cells, either untransfected or treated with siRNA(PLCβ1) for 48 h, were stimulated with carbachol or isoproterenol for Gαq or Gαi activation, respectively, or subjected to heat shock or hypo-osmotic stress, followed treatment with MG132 to prevent protein degradation before by lysis and western blotting where n = 3, * denotes p values between 0.01 and < 0.001 and SE is shown.

In Fig. 3A, we compare cytosolic levels of *ATP5f1b*, *Chgb* and *tau* under control, Gαq stimulation, heat shock, osmotic stress and Gαi stimulation. Compared to controls, we find the heat shock and osmotic stress significantly reduce the levels of all four genes subjected to stress, while levels of *ATP5f1b* and *Chgb* are protected upon Gαq activation. These data are consistent with specific protection from degradation or translation of *ATP5f1b* and *Chgb* in Ago2 stress granules formed upon Gαq activation. Activation of Gαi, increases *ATP5f1b* and *Chgb* and does not affect *tau*, consistent with its role in promoting growth pathways^19^.

### Impact of Gαq stimulation on ATP51b and Chgb proteins levels

We measured changes in the protein levels of Chgb and ATP5f1b in cells subjected to different environmental stress (Fig. 3B). Because changes in protein levels are subject to many post-synthetic modifications and regulatory mechanisms besides translation, we added other conditions to better assess the role of Gαq stimulation. MG132 proteasome inhibitor was used as a pre-treatment before stress, as the rate of our target protein turnover is short (~ 1 h)^20^ resulting in a lack of accumulation of protein when long term stress is applied. In one series of studies, PLCβ1 was down-regulated to promote Ago2 particle formation without Gαq activation^4^, and but this did not change protein levels. Additionally, no changes are seen in cells where Gαq was stimulated for 10 min or 30 min. However, heat shock, osmotic stress and stimulation of Gαi by isoproterenol all impact protein levels. The lack of change in Chgb and ATP5f1b with Gαq stimulation as opposed to other stress conditions suggests that the Gαq/PLCβ pathway only influences longer-term changes in mRNA through Ago2 stress granules and not short-term protein levels.

**Figure 4:**
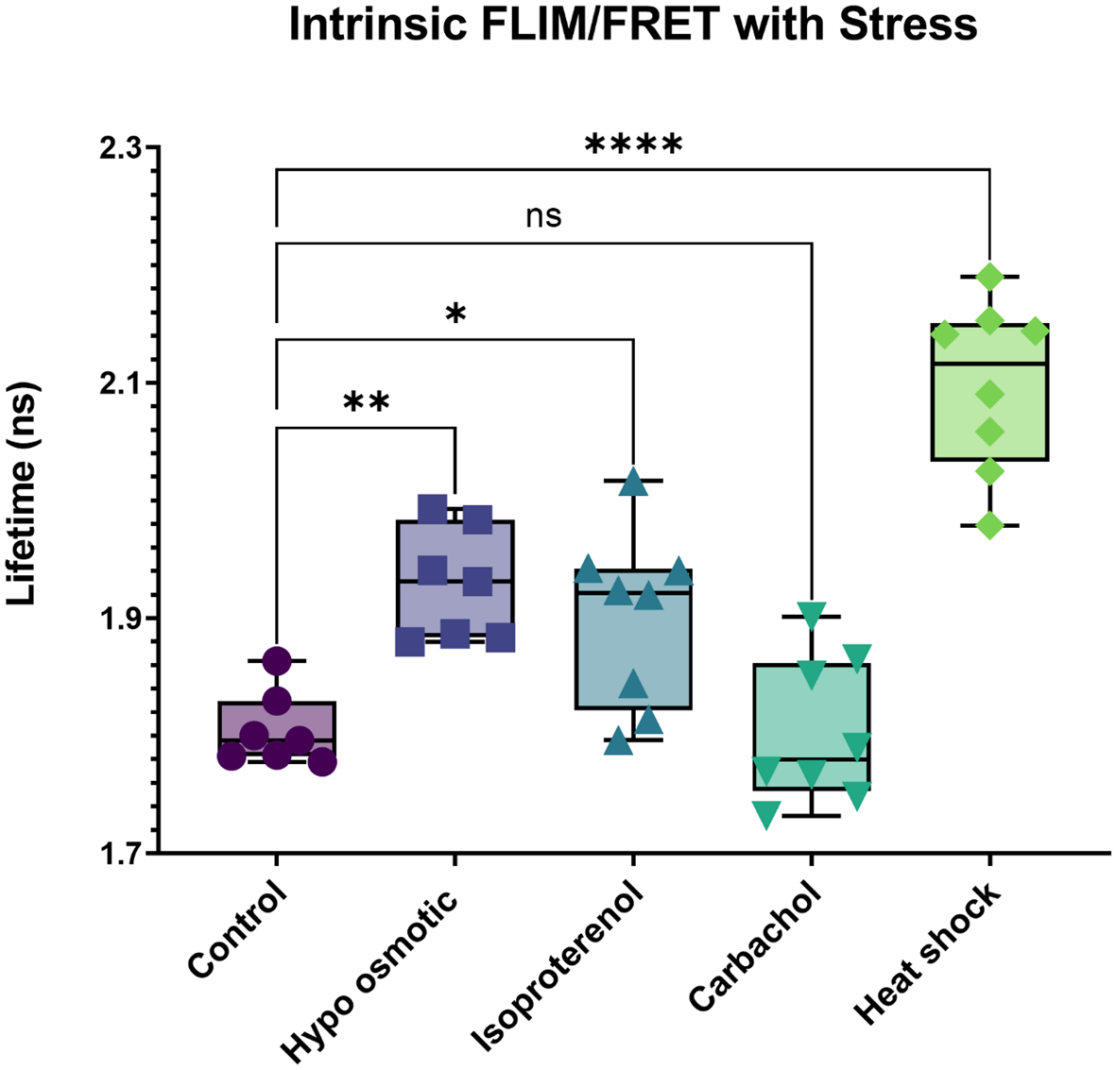
Intrinsic FLIM measurements to evaluate mitochondrial health. Untransfected, differentiated PC12 cells were exposed to carbachol, osmotic stress and heat shock before imaging at 740 nm excitation using a two photon laser, and measuring the autofluorescence lifetime by phase-modulation where n = 8. P values were calculated using one-way ANOVA analysis comparing the specific sample with control. * = p ≤ 0.05, ** = p ≤ 0.01, *** = p ≤ 0.001, **** = p ≤ 0.0001.

In a final series of studies, we assessed the protection of ATP5f1b with Gαq stimulation, by monitoring changes in the redox state of cells to assess mitochondria health and ATP production. These experiments measured changes in intrinsic fluorescence contributed mainly by NAD(P)H and FADH reflecting the optical redox state of mitochondria (see^21^). NADH, which is generated through the Krebs cycle, has a longer-lived fluorescence compared to other intrinsic fluorophores (see^22^). We measured the intrinsic fluorescence of cells under different stress conditions and found that while the lifetime of cells treated with carbachol to activate Gαq is the same as control, cells subjected to osmotic stress, isoproterenol or heat trend towards higher values (Fig. 4). This higher lifetime is consistent with a transfer of ATP production to the Krebs cycle in response to reduced ATP synthase activity. These results support the idea that ATP5fb1 levels are protected with Gαq stimulation in contrast to other stresses.

## Discussion

Mammalian Gαq and its analogs in other species play a key role in generating cellular responses by increasing levels of intracellular calcium^19^. In this study, we have uncovered a novel pathway in which Gαq may control the translation of specific mRNAs. Our studies show that in differentiated PC12 cells, Gαq activation sequesters two mRNAs important for endocrine function and cellular energy in Ago2 stress granules.

The basis of this pathway stems from the multifunctional nature of PLCβ1. PLCβ1 has several conserved structural domains surrounding the catalytic domain along with a long 400aa tail that is required for Gαq binding, and that can mediate binding to cytosolic partners (see^23^). One of these confirmed partners is the Promoter of RNA-induced silencing, C3PO^24,25^, which is inhibited by PLCβ1 binding^3,26–28^. In recent work, we characterized the proteins bound to cytosolic PLCβ1 in undifferentiated PC12 cells and found roughly 30% of bound proteins are identified as stress granules proteins, including Ago2, PABPC, eIF5a and G3BP1^4,29^. These studies showed that reducing the cytosolic level of PLCβ1 either by down-regulation, osmotic stress or Gαq activation allowed for the release of bound proteins and promoted the formation of stress granules. While stress granules protect mRNA from degradation during stress conditions^5^, it is surprising these particles form under routine physiological responses generated by Gαq. This observation prompted us to determine whether the stress granules formed upon Gαq activation had a specific physiological impact on cells.

Our previous studies using undifferentiated PC12 cells and two muscle cell lines, A10 and WKO-3M22, showed that Gαq activation promotes the assembly of stress granules as indicated by established markers, G3BP1 and PABPC, as well as Ago2^4^ (see^15,30–33^). Those studies identified Ago2 as either a direct or indirect binding partner of PLCβ1 by pull down studies and by FRET using tagged proteins, and showed Ago2 particles that grew in size and number under various stress conditions. Here, we show similar stress granule assembly as followed by the marker protein G3BP1 and that these particles are stable for at least 30 min under our conditions.

Formation of stress granules depends not only on the environmental conditions, but also on the nature of the bound miRs and mRNA^8,12,32,34^, and we reasoned that the binding of these species may be influenced by proteins that reside in stress granules. To this end, we characterized the proteins associated with Ago2 stress granules using a proteomics approach. Additionally, because previous studies suggested that different stresses form stress granules with distinct composition^30^, here we identified proteins in Ago2-associated stress granules in cells subjected to Gαq stimulation, osmotic stress and heat. We find that the shifts in composition were surprisingly minor especially considering their different cumulative responses. All stress conditions found that TEF-5 is a major binding partner of Ago2. TEF-5 is a transcriptional enhancer factor that works through the hippo pathway to inhibit proliferation^35^, and its association with Ago2 may serve to regulate TEF-5 translation to the nucleus. Comparing the top ten proteins in the Ago2 proteomics data sets, we find only one unique protein for Gαq stimulation, polyubiquitin-B, which impacts protein turn-over as well as various signaling cascades^36^. It is notable that stress granules formed with carbachol stimulation have a higher percentage of calcium-sensitive proteins.

Under control conditions, we find that Ago2 is associated with many proteins but these complexes do not contain RNA meaning that they are not true stress granules. The observation that only two major transcripts (*ATP5f1b* and *Chgb*) are sequestered in Ago2 transcripts formed in response to Gαq stimulation was surprising. Our N&B studies found ~ 40% of G3BP1 aggregated with carbachol stress but we note that only 30% of the cells responded. By comparing the amount by total cellular *ATP5f1b* with the amount isolated in Ago2 particles per cell as determined by PCR where there was little loss in total Ago2, we find that at least 5% of this mRNA transcript is contained in stress granules. However, this value does not account for loss of RNA in the RNAseq measurements, and because our data show complete protection of this transcript, we suspect the value is much higher. It is important to note that we also find that cells recover very little after the stress is removed for 30 min implying a relatively long-term protection of these transcripts. Thus, if a larger cell population responds by increasing or repeating stimulation, then a much higher percent of the transcripts will be protected. By this argument, repeated stress would cause a significant population of transcripts to accumulate in Ago2 stress granules impacting cell function.

Maintaining cellular energy production during and after signaling is important, and the functional impact of preserving *ATP5f1b* during Gαq stimulation is seen though the preservation of the redox state (Fig. 4). Similarly, Gαq is found in many cell types with endo- and exocrine function, and preservation of Chga/b, which mediates vesicle formation, is expected. Of course, these studies focused on differentiated PC12 cells that have secretory function^14^, but it is very likely that other cell types will sequester other mRNAs upon Gαq stimulation.

**Figure 5:**
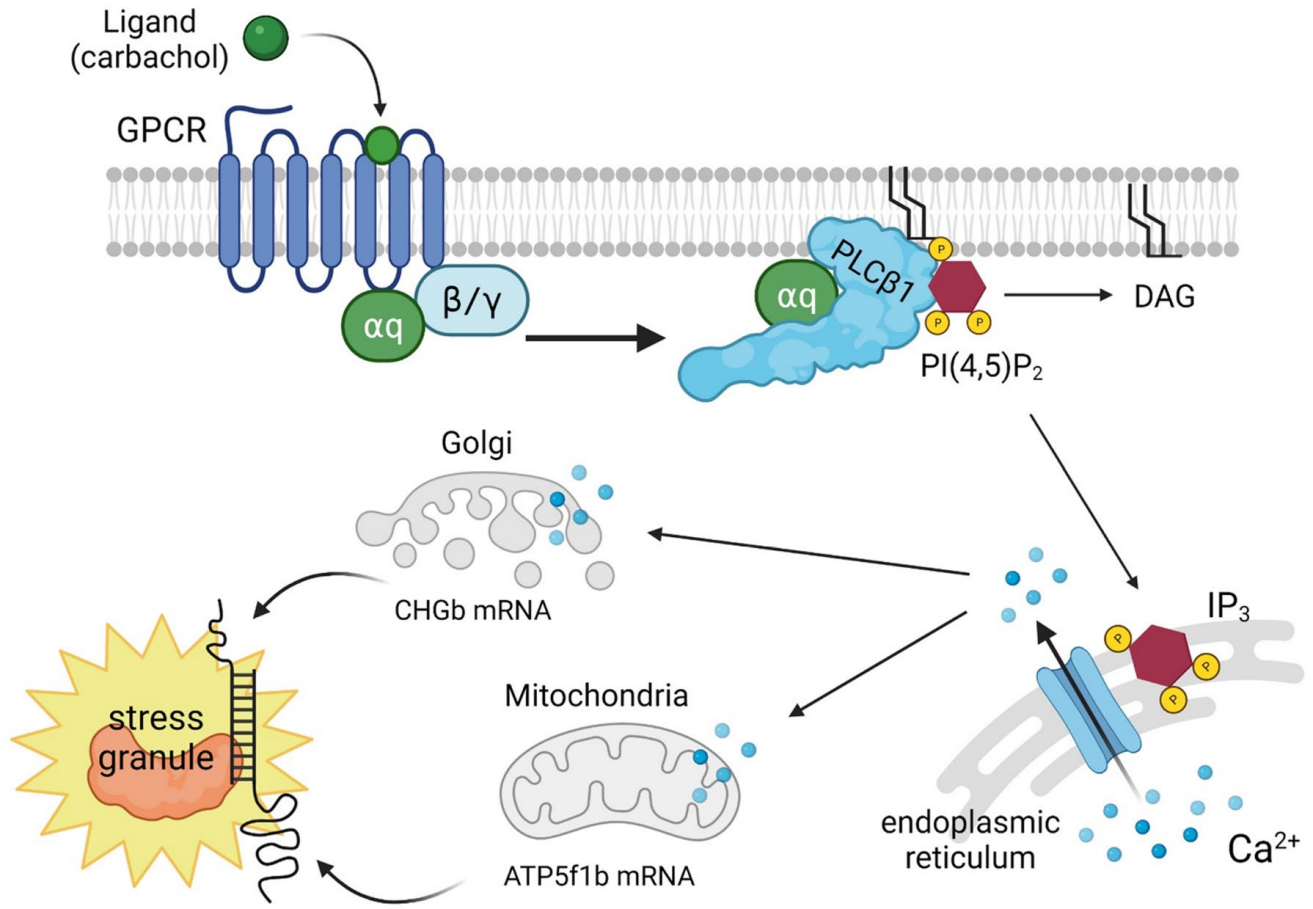
Model connecting the Gαq pathway with specific transcripts sequestered with activation. When PLCβ1 is activated by Gαq, Ca^2+^ is released. This excess calcium is believed to flood the mitochondria and Golgi, leading to the movement of *Chga/b* and *ATP5f1b* mRNA into stress granules.

The selection of these two specific transcripts by Ago2 stress granules is unclear and is under investigation. Because Gαq stimulation results in an increase in intracellular calcium, it is possible that distinct calcium sensitive proteins contained in the Ago2 stress granules that form, are directly or indirectly responsible for specifically recruiting these transcripts. A model connecting Gαq activation and the two transcripts sequestered in the Ago2 stress granules formed is shown in Fig. 5.

PLCβ1 is associated with loss of proliferation and differentiation^37,38^ and these effects are likely to be due to its interaction with C3PO and Ago2, as well as mediating calcium signals. We have previously identified miRs associated PLCβ1 down-regulation in PC12 cells^39^ and found that down-regulating PLCβ1 levels results in substantial reduction in miR101a and miR101b, which may regulate ATP synthase 5B^40^and miR-22 levels by 20%, which regulates *Chgb*^41^. The loss of these miRs, along with the observation that loss of cytosolic PLCβ1 or sustained activation of Gαq returns PC12 cells to the undifferentiated state, may indicate that the increased number of stress granules may sequester mRNAs important for defining cell phenotype.

## Methods

### Cell culture

PC12 cells were cultured in high glucose DMEM (Gibco) with 10% heat inactivated horse serum (Gibco), 5% fetal bovine serum (Atlanta Biologicals) and 1% Penicillin/Streptomycin (Gibco). Cells were treated with 0.1 ug/mL nerve growth factor (Bon Opus) for ~ 48 h in media containing 1% heat inactivated horse serum and 1% antibiotic to induce differentiation. All cells were incubated at 37 °C in 5% CO_2_. Cells below passage 10 were used for all experiments.

### Plasmids

EGFP-hArgonaute2 (eGFP-Ago2) was purchased from Addgene (plasmid # 21981) and was prepared in the laboratory of Philip Sharp (MIT). G3BP1-GFP was purchased from Addgene (plasmid # 119950) and was prepared in the laboratory of Jeffrey Chao (Friedrich Miescher Institute). siRNA(PLCβ1) was from Dharmacon (smartpool # L-092936-02-0005). Plasmid transfections and siRNA knockdowns were done using Lipofectamine 3000 (Invitrogen) in antibiotic free media. Medium was changed to one containing antibiotic (1% Penicillin/Streptomycin) 4–12 h post-transfection.

### Stress conditions

Cells were exposed to carbachol or isoproterenol stimulation at a final concentration of 5 µm for 10 min. For hypo-osmotic stress, the culture medium was diluted from 300 to 150 mOsm with 50% water for 5 min. For heat shock, cells were incubated at 42 °C for 1 h. Pertussis toxin (Invitrogen, cat # PHZ1174) was incubated in cell media at a concentration of 100 ng/mL for 24 h before applying 10 min carbachol stimulation.

### Number and brightness (N&B) measurements

N&B defines the number of photons associated with a diffusing species by analyzing the variation of the fluorescence intensity in each pixel in the cell image, and full details about the method and analysis can be found in^16^. In these studies, we collected ~ 100 cell images viewing either free eGFP (control) or eGFP-G3BP1 at a 66 nm/pixel resolution and at a rate of 4 µs/pixel (see^4^). Images obtained were 256 × 256 pixels. The size of the SimFCS4 boxes used was determined by the amount of monomeric protein seen in unstressed controls. Any pixels on the B vs I plot that were outside of this region were determined to be protein aggregation due to the stress applied. Percent aggregation was calculated based off of total number of pixels outside of the control (red box) region, divided by the total number of pixels in the cell. The percent aggregation corresponding to either oligomerization (pixels contained in the green box) or aggregated monomeric protein (pixels contained in the blue box) are also reported using text in the respective color. N&B samples were imaged on dual-channel confocal (Alba version 5, ISS Inc.) using a two-photon titanium-sapphire laser and a Nikon Eclipse Ti-U inverted microscope as described^39^. All measurements were taken at 850 nm wavelength using RICS/N&B mode on VistaVision software. Samples were imaged at room temperature. Number and Brightness (N&B) data were analyzed with SimFC4 software (www.lfd.uci.edu) where the brightness, B, in each pixel refers to the ratio of the variance, σ, over the average fluorescence intensity < k > :

$$B= \sigma 2/<k>$$

and

$$<k>= \epsilon n$$

where n is the number of fluorophores. The variance in each pixel is obtained by rescanning the cell image ~ 100 times. The average fluorescence intensity, < k > , is directly related to the molecular brightness, ε, in units of photons per second per molecule. Data taken at various sampling times (50 frames/30 s, vs 200 frames/90 s) were compared, and yielded identical results.

### Particle analysis

Samples were imaged with a 100X/1.49 oil TIRF objective to microscopically count the number of particles per µm^2^ formed under different conditions. For each condition, 10 to 20 cells were randomly selected, and z-stack measurements were taken (1.0 µm/frame). Analysis was performed with ImageJ and Fiji ImageJ software either by thresholding before analyzing, and averaging the number of particles per frame per measurement or by combining z-stack measurements to generate a 3D image for each sample before analyzing the number of particles per sample and averaging the results. Both methods produced identical results.

### Mass spectrometry

Ago2 stress granules were purified from PC12 cells electroporated with eGFP-Ago2 then differentiated with NGF for ~ 48 h using the protocol described by Wheeler et al*.*^42^. Six 150 mm dishes of cells were used per condition. Immunoprecipitation was done using GFP antibody (Santa Cruz Biotechnology, sc-9996) with protein A dynabeads and elution buffer (Invitrogen,cat #10006D). Following short gel electrophoresis and band excision, mass spectrometry was performed at UMass Medical School Mass Spectrometry Facility. Bound proteins were reported using Scaffold software. Each protein in this report had a minimum of one total spectrum count measured for at least one condition.

### eCLIP transcript sequences

PC12 cells were transfected with eGFP-Ago2 using electroporation (Bio-Rad Gene Pulser Xcell) with one pulse in a 0.4 cm gap cuvette (Bio-Rad, cat #1652088) in complete media and plated at ~ 50% confluency to leave room for neurite growth. Cells were stressed and crosslinked using 254 nm ultraviolet light. Treated cells were collected from at least four 150 mm culture dishes per sample condition. Lysis was done on fresh, non-frozen cell pellets using 100 µL lysis buffer supplemented with RNase inhibitor and protease inhibitor tablets (Roche). A 10 µL portion of each lysate was used to extract total RNA (Zymo Clean and Concentrator) which was then tested for RNA integrity using RNA Fragment Analyzer services at University of Massachusetts Medical School Molecular Biology Core Lab. The remaining lysate was immunoprecipitated using mouse Ago2 antibody. Subsequent western blots, RNA cleanup and library construction was done using Eclipse Bioinnovation protocol v1.01R, storing samples at − 80 °C overnight when necessary. Sample quantity was evaluated by qPCR. Libraries were sequenced at UMass Medical School Deep Sequencing Core using Illumina HiSeq 4000 system. All reagents were supplied by Eclipse Bioinnovations unless otherwise specified.

### RT-PCR

For each condition, one 100 mm dish was used for total RNA extraction. Cells were differentiated and stressed before isolating RNA using RNeasy Mini Kit (Qiagen, Cat #74104) with DNase digestion (Qiagen, cat #79254). CDNA synthesis was done using MiniAmp Thermal Cycler (Applied Biosystems). Purified RNA sample concentrations were measured by Nanodrop and 1 µg RNA was incubated with dNTP (New England Biolabs, cat #N0447S), random hexamers (Thermo Scientific, cat #SO142), and nuclease free water (Invitrogen, cat #AM9930), at 65 °C for 5 min. After the first incubation, 5 × First Strand Buffer, DTT (Invitrogen cat #18080093) and RNase inhibitor (Invitrogen, cat #AM2694) were added to the mixture and incubated at 25 °C for 2 min. After the second incubation, 1 µL Superscript III Reverse Transcriptase (Invitrogen, cat #18080093) was added and the mixture was incubated at 25 °C for 10 min, 42 °C for 50 min, and 70 °C for 15 min. CDNA was stored at − 20 °C overnight or used immediately at a dilution of 1:20 in nuclease free water for RT-PCR experiments with Luna Universal Master Mix (New England Biolabs, cat #M3003L), nuclease free water, and custom ATP5f1b and CHGb primers (ITD). For positive and negative controls, 18S ribosomal subunit and tau primers were used (see Supplemental).

### Western blotting

We used an establish procedure^39^ with modifications. Samples were grown in 100 mm dishes plates and were treated with MG132 at a final concentration of 250 nM 24 h before collection. Cells were stressed and then collected in 250 μl of NP-40 lysis buffer [150 mM NaCl, 50 mM Tris (pH = 8.0), 1% NP40, protease inhibitor]. The primary antibodies used included: anti-ATP5f1b (Abcam, ab117991), anti-Chgb (Abcam, ab150354), anti-actin (Santa Cruz, sc-47778).

### Fluorescence lifetime imaging measurements (FLIM)

Phase modulation FLIM measurements were performed on the dual-channel confocal fast FLIM (Alba version 5, ISS Inc.) using a two-photon titanium-sapphire laser and a Nikon Eclipse Ti-U inverted microscope as described^39^. The lifetime of the laser was calibrated each time before experiments by measuring the lifetime of Atto 425 in water with a lifetime of 3.61 ns at 80, 160, and 240 MHz. The samples were excited at 740 nm for intrinsic measurements, and emission spectra were collected through a 525/50 bandpass filter. For each measurement, the data were acquired using FastFLIM mode on VistaVision software until the photon count was > 100.

### Statistical analysis

Data were analyzed with the Sigma Plot 14.5 and Graphpad Prism 9 statistical packages, which included the student’s *t* test and one-way analysis of variance (ANOVA).

## Supplementary Information

Supplementary Information 1.

Supplementary Information 2.

Supplementary Information 3.

Supplementary Information 4.

Supplementary Information 5.

Supplementary Information 6.

Supplementary Information 7.

## Data Availability

The datasets used and/or analysed during the current study available from the corresponding author on reasonable request.
