# Author Correction: Activation of Gαq sequesters specific transcripts into Ago2 particles

**DOI:** 10.1038/s41598-022-16292-2

**Published:** 2022-07-11

**Authors:** Lela Jackson, Madison Rennie, Alison Poussaint, Suzanne Scarlata

**Affiliations:** Department of Chemistry and Biochemistry, Worcester Polytechnic Institute, 100 Institute Rd, Worcester, MA 01609 USA

Correction to: *Scientific Reports* https://doi.org/10.1038/s41598-022-12737-w, published online 24 May 2022

The Funding section in the original version of this Article was omitted.

The Funding section now reads: “This work was supported by funding from the Richard Whitcomb foundation. Alison Poussaint was supported by NSF-REU CHE-1950512”.

The original Article has been corrected.

