# Supplementary material for: Activation of Gαq sequesters specific transcripts into Ago2 particles
Source: Sci Rep. 2022 May 24;12:8758. doi: 10.1038/s41598-022-12737-w (PMC9130320; doi:10.1038/s41598-022-12737-w)

ATP5f1b showing edges (25 kDa)

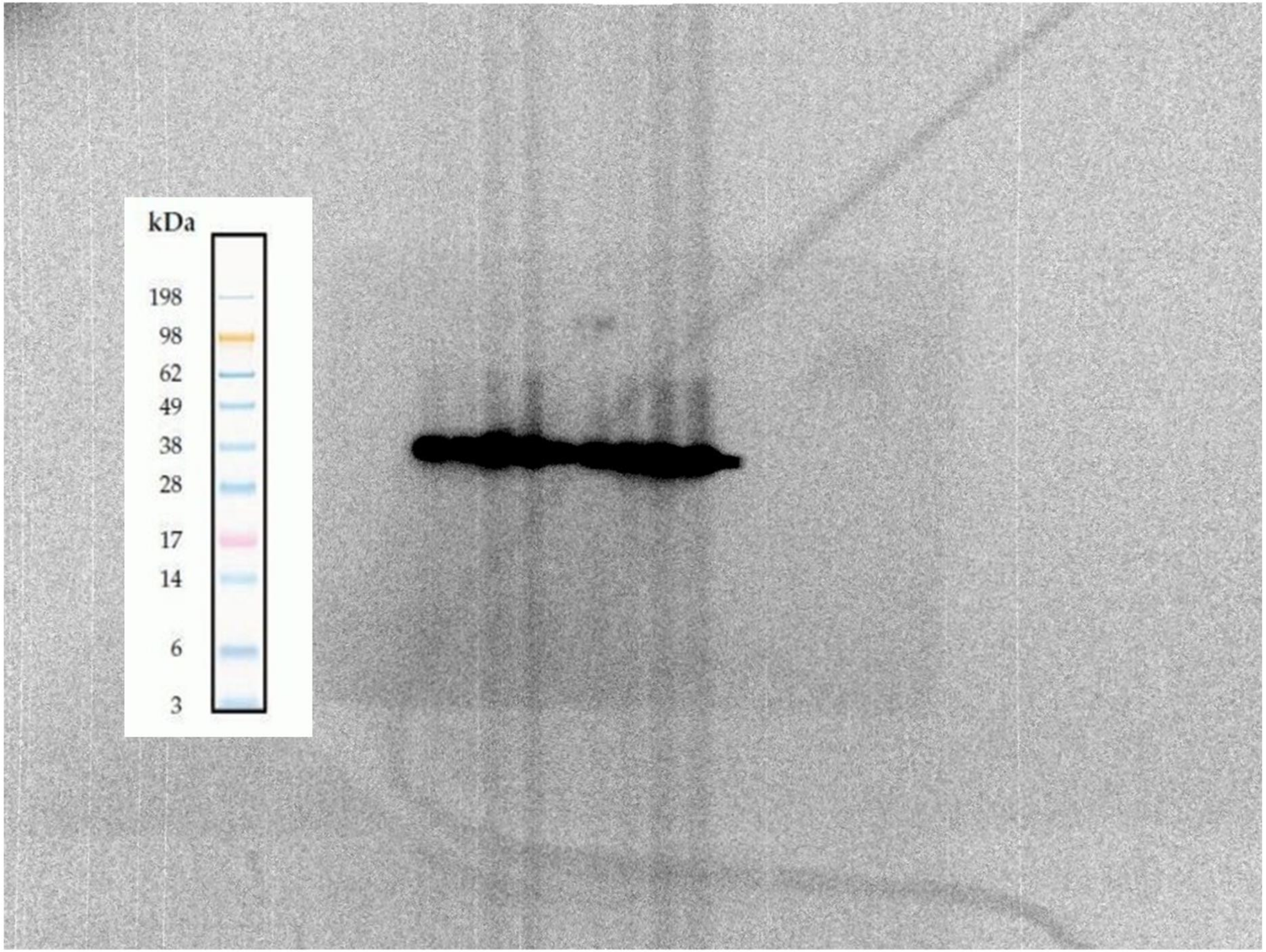

ATP5f1b (25 kDa)

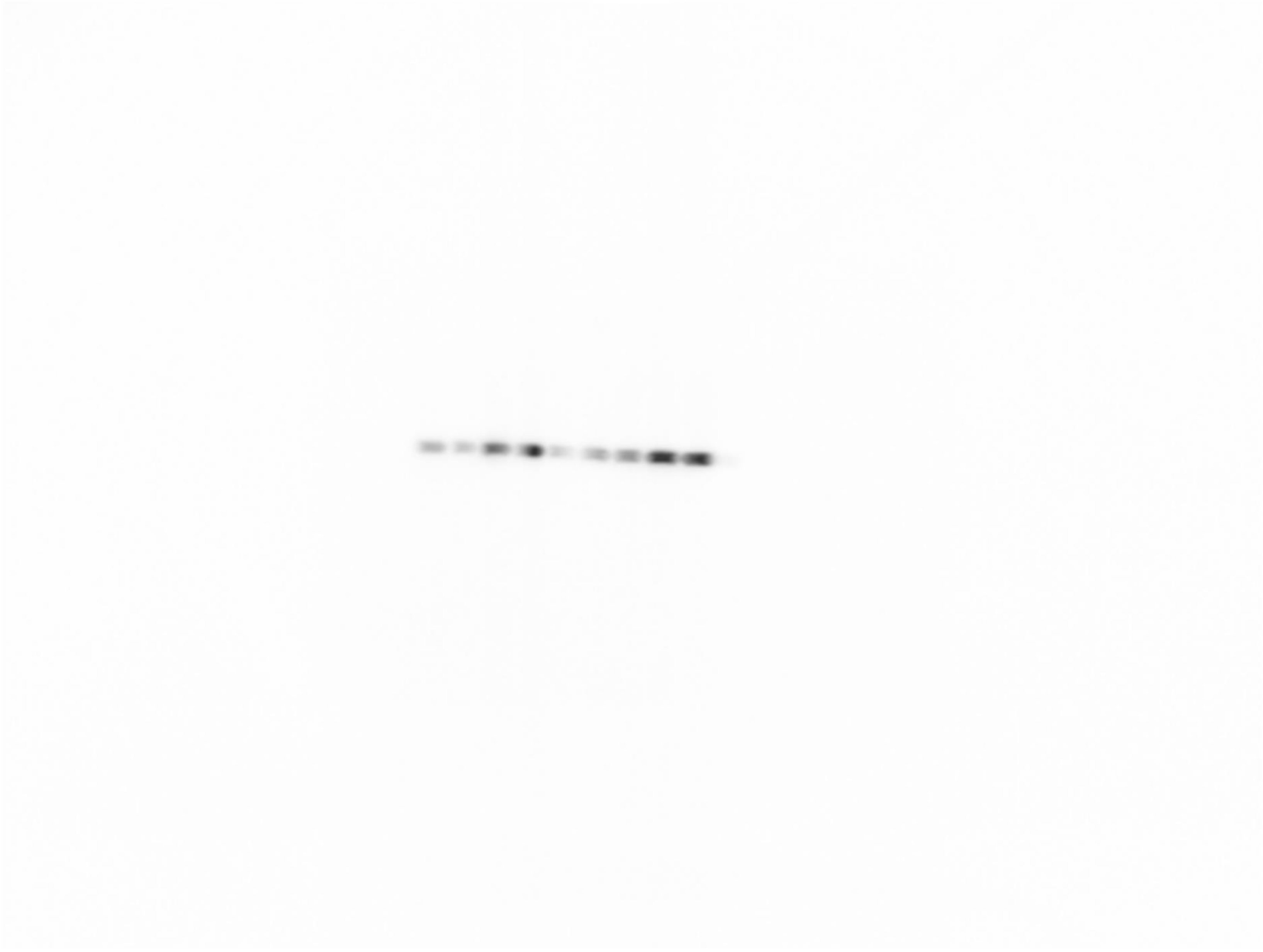

CHGb showing edges (78 kDa)

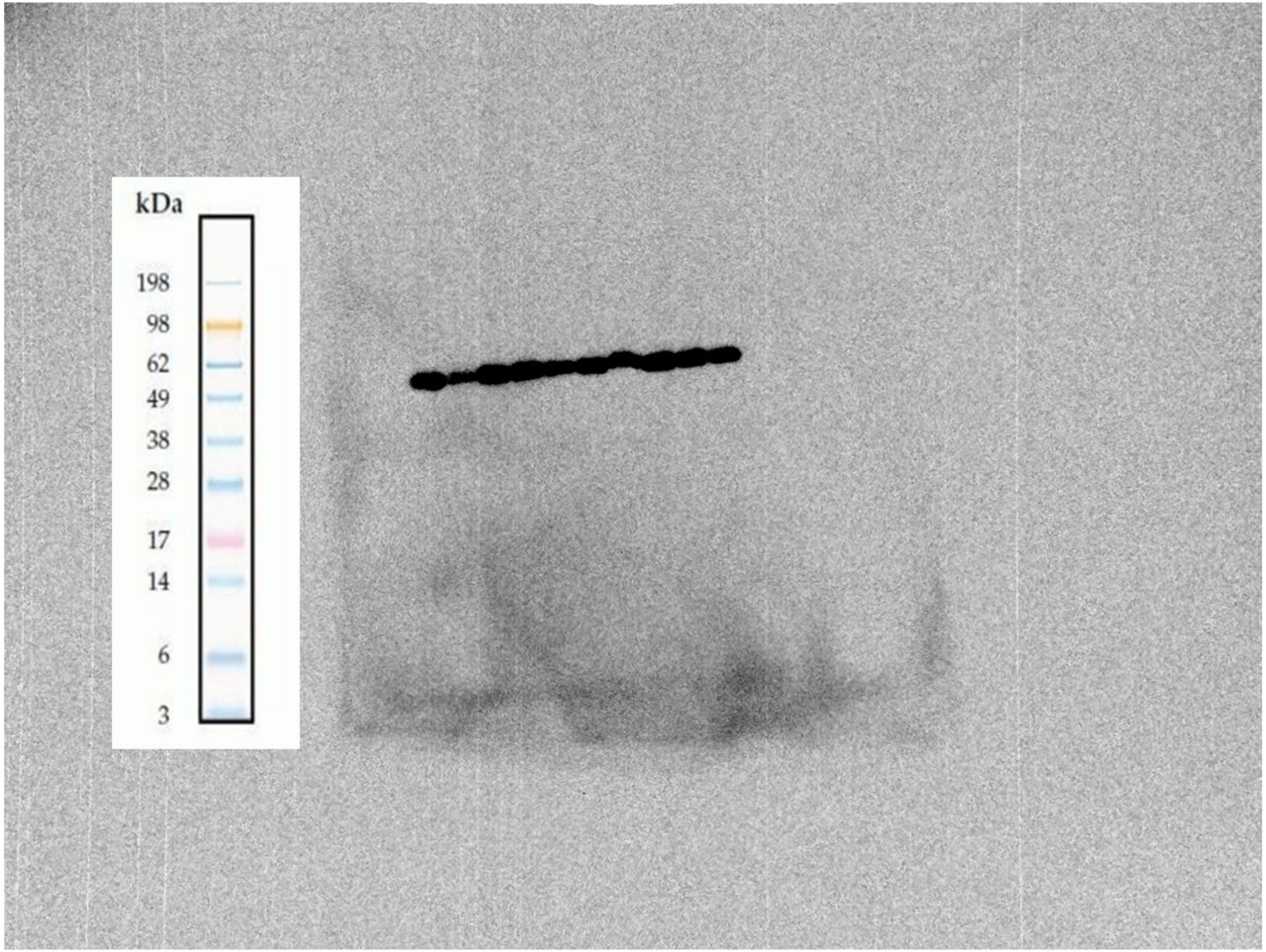

CHGb (78 kDa)

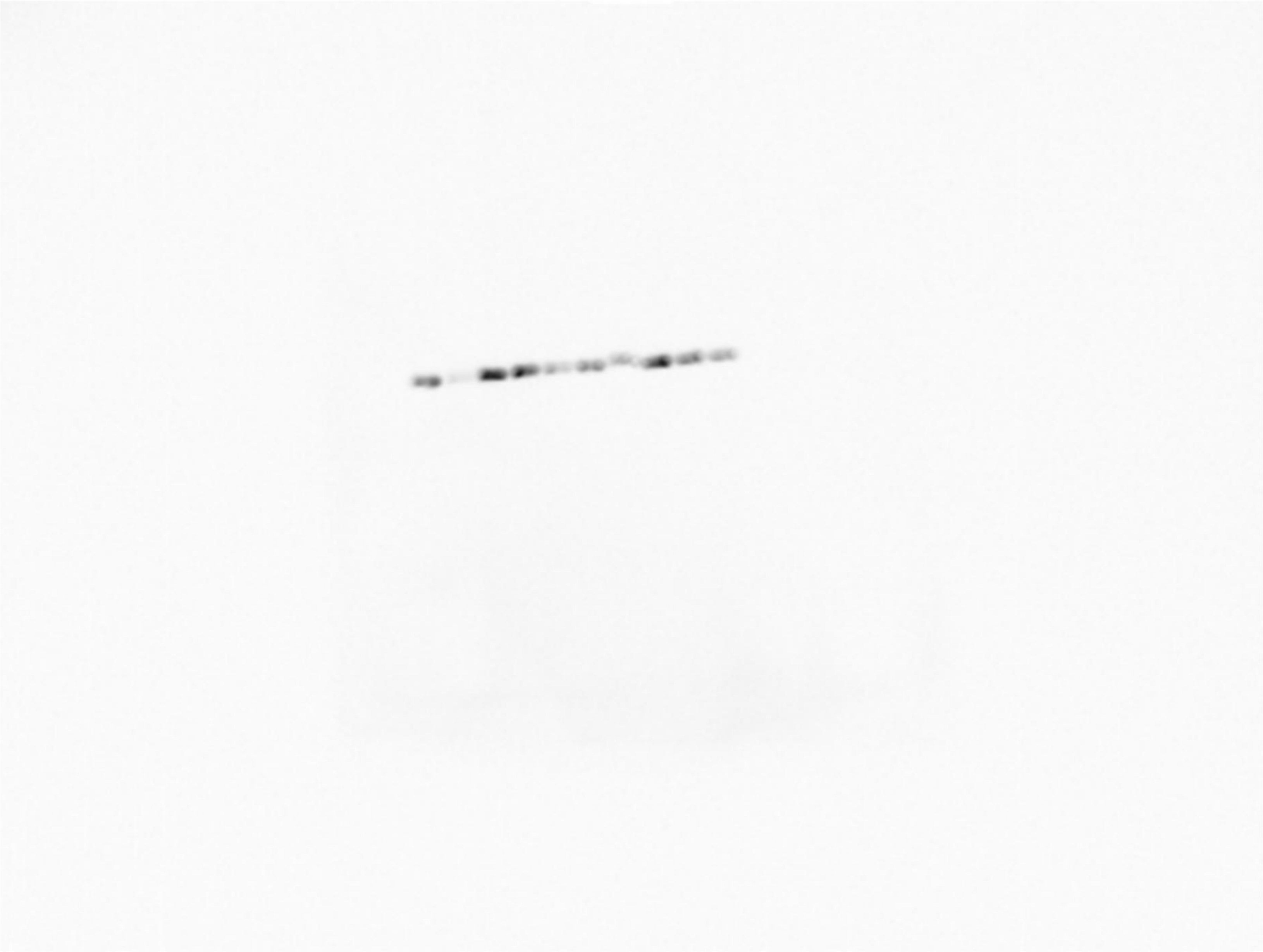

Actin showing edges (42 kDa)

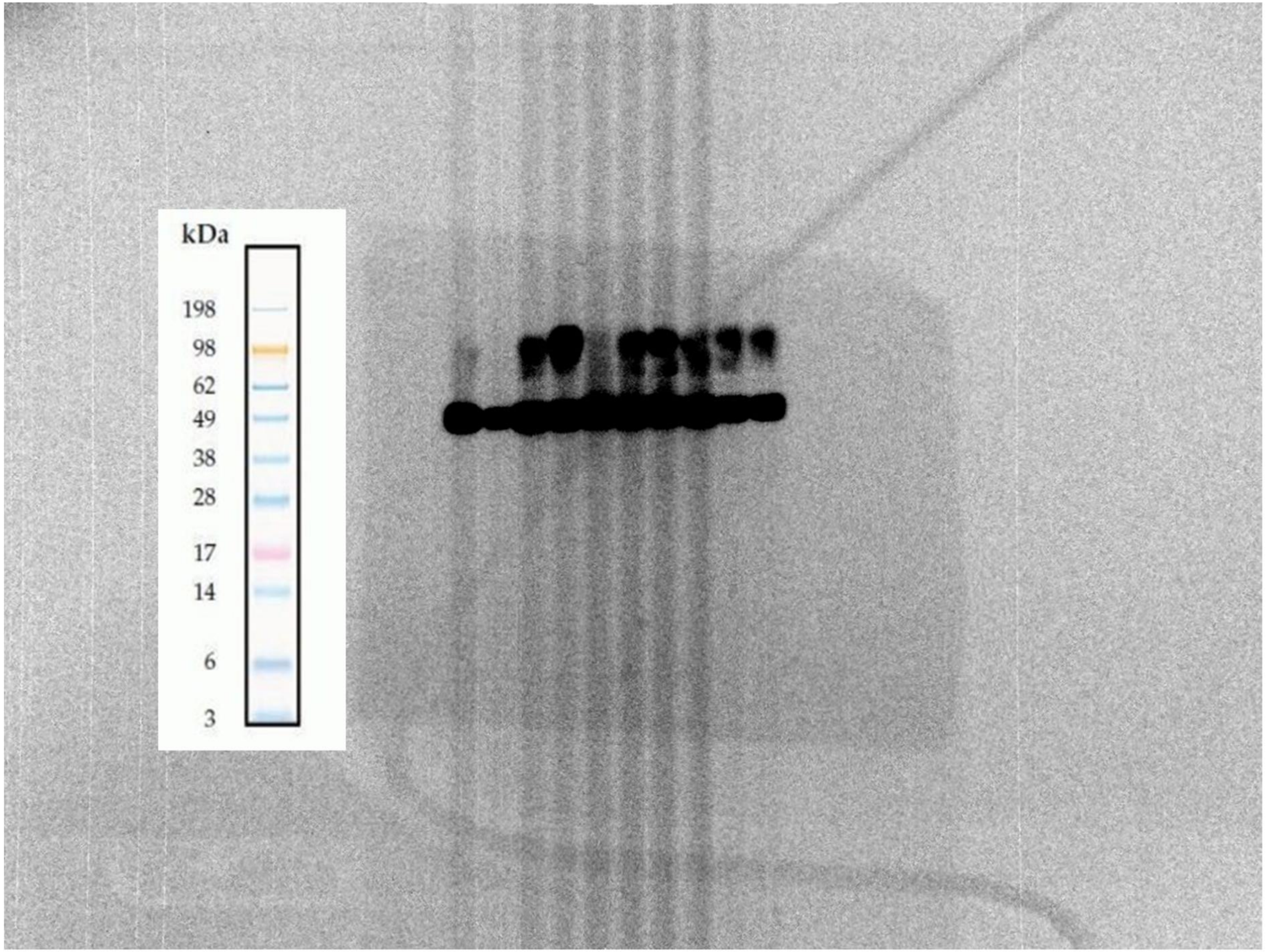

Actin (42 kDa)

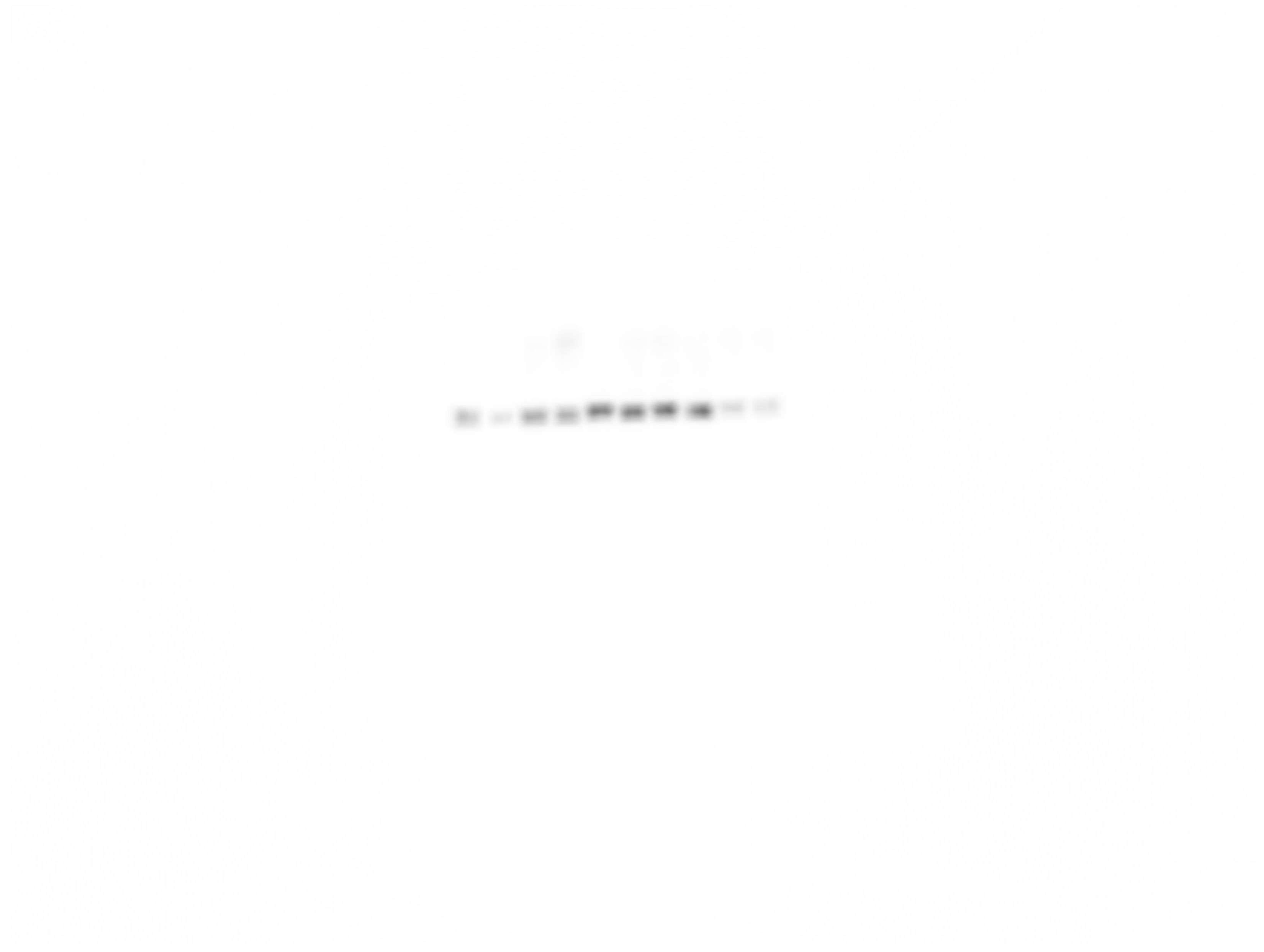

Supplement: Supplementary file 1 — Supplementary Information 1. [file 41598_2022_12737_MOESM1_ESM.pdf]
