# Supplementary material for: Activation of Gαq sequesters specific transcripts into Ago2 particles
Source: Sci Rep. 2022 May 24;12:8758. doi: 10.1038/s41598-022-12737-w (PMC9130320; doi:10.1038/s41598-022-12737-w)
Supplement: Supplementary file 2 — Supplementary Information 2. [file 41598_2022_12737_MOESM2_ESM.pdf]

SI Table 1 Proteins bound to Ago2 in cells under basal conditions

CONTROL SPECIFIC

mRNA:

sp|Q3ZAV8|EDC4_RAT Enhancer of mRNA-decapping protein 4 OS=Rattus norvegicus OX=10116 GN=Edc4 PE=1 SV=1	sp|Q3ZAV8|EDC4_RAT (+2)	Edc4	153 kDa	99% (1)
tr|A0A091DGJ7|A0A091DGJ7_FUKDA mRNA-capping enzyme (Fragment) OS=Fukomys damarensis OX=885580 GN=H920_09191 PE=4 SV=1	tr|A0A091DGJ7|A0A091DGJ7_FUKDA (+6)	H920_09191	53 kDa	99% (1)
tr|A0A091DJ53|A0A091DJ53_FUKDA Pre-mRNA-splicing factor 38B OS=Fukomys damarensis OX=885580 GN=H920_15753 PE=4 SV=1	tr|A0A091DJ53|A0A091DJ53_FUKDA (+3)	H920_15753	59 kDa	99% (1)
tr|A0A1S3EQY6|A0A1S3EQY6_DIPOR regulation of nuclear pre-mRNA domain-containing protein 1B isoform X2 OS=Dipodomys ordii OX=10020 GN=Rprd1b PE=4 SV=1	tr|A0A1S3EQY6|A0A1S3EQY6_DIPOR (+3)	Rprd1b	35 kDa	99% (1)
tr|A0A1S3FCW4|A0A1S3FCW4_DIPOR pre-mRNA-processing factor 40 homolog B isoform X2 OS=Dipodomys ordii OX=10020 GN=Prpf40b PE=4 SV=1	tr|A0A1S3FCW4|A0A1S3FCW4_DIPOR (+2)	Prpf40b	98 kDa	99% (1)

Transcription factor:

tr|A0A091CZI5|A0A091CZI5_FUKDA General transcription factor 3C polypeptide 2 OS=Fukomys damarensis OX=885580 GN=H920_15241 PE=3 SV=1	tr|A0A091CZI5|A0A091CZI5_FUKDA	H920_15241	162 kDa	100% (2)
tr|A0A0G2K7N8|A0A0G2K7N8_RAT Bromodomain PHD finger transcription factor OS=Rattus norvegicus OX=10116 GN=Bptf PE=1 SV=1	tr|A0A0G2K7N8|A0A0G2K7N8_RAT	Bptf	304 kDa	100% (2)
tr|A0A0P6JTL2|A0A0P6JTL2_HETGA HMG box transcription factor BBX isoform 1 OS=Heterocephalus glaber OX=10181 GN=BBX PE=4 SV=1	tr|A0A0P6JTL2|A0A0P6JTL2_HETGA (+1)	BBX	105 kDa	99% (2)
sp|F1LMN3-2|E2F8_RAT Isoform 2 of Transcription factor E2F8 OS=Rattus norvegicus OX=10116 GN=E2f8	sp|F1LMN3-2|E2F8_RAT (+2)	E2f8	94 kDa	99% (1)
sp|P0C6P6|TYY2_RAT Transcription factor YY2 OS=Rattus norvegicus OX=10116 GN=Yy2 PE=3 SV=1	sp|P0C6P6|TYY2_RAT (+4)	Yy2	42 kDa	99% (1)
sp|P46153|GATA6_RAT Transcription factor GATA-6 OS=Rattus norvegicus OX=10116 GN=Gata6 PE=1 SV=2	sp|P46153|GATA6_RAT (+1)	Gata6	59 kDa	99% (1)
sp|P70486|ATRX_RAT Transcriptional regulator ATRX (Fragment) OS=Rattus norvegicus OX=10116 GN=Atrx PE=1 SV=1	sp|P70486|ATRX_RAT	Atrx	59 kDa	99% (1)
sp|Q4QR87|DMRTC_RAT Doublesex- and mab-3-related transcription factor C1 OS=Rattus norvegicus OX=10116 GN=Dmrtc1 PE=2 SV=1	sp|Q4QR87|DMRTC_RAT	Dmrtc1	24 kDa	99% (1)
sp|Q561Q8|MED4_RAT Mediator of RNA polymerase II transcription subunit 4 OS=Rattus norvegicus OX=10116 GN=Med4 PE=2 SV=1	sp|Q561Q8|MED4_RAT (+3)	Med4	30 kDa	99% (1)
sp|Q66HG1|DMTF1_RAT Cyclin-D-binding Myb-like transcription factor 1 OS=Rattus norvegicus OX=10116 GN=Dmtf1 PE=2 SV=2	sp|Q66HG1|DMTF1_RAT (+4)	Dmtf1	84 kDa	99% (1)
sp|Q6AXU9|CNOT6_RAT CCR4-NOT transcription complex subunit 6 OS=Rattus norvegicus OX=10116 GN=Cnot6 PE=2 SV=1	sp|Q6AXU9|CNOT6_RAT (+5)	Cnot6	63 kDa	99% (1)
tr|A0A091CQ14|A0A091CQ14_FUKDA Transcription initiation factor TFIID subunit 7 OS=Fukomys damarensis OX=885580 GN=H920_19286 PE=4 SV=1	tr|A0A091CQ14|A0A091CQ14_FUKDA (+4)	H920_19286	40 kDa	99% (1)
tr|A0A091CQ91|A0A091CQ91_FUKDA Transcription factor NF-E2 45 kDa subunit OS=Fukomys damarensis OX=885580 GN=H920_17342 PE=3 SV=1	tr|A0A091CQ91|A0A091CQ91_FUKDA (+1)	H920_17342	41 kDa	99% (1)
tr|A0A091CYB9|A0A091CYB9_FUKDA Mediator of RNA polymerase II transcription subunit 11 OS=Fukomys damarensis OX=885580 GN=MED11 PE=3 SV=1	tr|A0A091CYB9|A0A091CYB9_FUKDA	MED11	13 kDa	99% (1)
tr|A0A091D225|A0A091D225_FUKDA Signal transducer and activator of transcription OS=Fukomys damarensis OX=885580 GN=H920_14054 PE=3 SV=1	tr|A0A091D225|A0A091D225_FUKDA	H920_14054	96 kDa	99% (1)
tr|A0A091D7D7|A0A091D7D7_FUKDA Pre-B-cell leukemia transcription factor 2 OS=Fukomys damarensis OX=885580 GN=H920_19927 PE=4 SV=1	tr|A0A091D7D7|A0A091D7D7_FUKDA	H920_19927	36 kDa	99% (1)
tr|A0A091D8Y4|A0A091D8Y4_FUKDA Transcription elongation factor A N-terminal and central domain-containing protein OS=Fukomys damarensis OX=885580 GN=H920_19321 PE=4 SV=1	tr|A0A091D8Y4|A0A091D8Y4_FUKDA	H920_19321	40 kDa	99% (1)
tr|A0A091DJB3|A0A091DJB3_FUKDA Transcription initiation factor TFIID subunit 13 OS=Fukomys damarensis OX=885580 GN=H920_07981 PE=4 SV=1	tr|A0A091DJB3|A0A091DJB3_FUKDA	H920_07981	13 kDa	99% (1)
tr|A0A091DWM1|A0A091DWM1_FUKDA Leucine-zipper-like transcriptional regulator 1 OS=Fukomys damarensis OX=885580 GN=H920_03876 PE=4 SV=1	tr|A0A091DWM1|A0A091DWM1_FUKDA (+1)	H920_03876	162 kDa	99% (1)
tr|A0A091DXE6|A0A091DXE6_FUKDA Transcription factor MafG OS=Fukomys damarensis OX=885580 GN=H920_02946 PE=3 SV=1	tr|A0A091DXE6|A0A091DXE6_FUKDA	H920_02946	25 kDa	99% (1)
tr|A0A091E162|A0A091E162_FUKDA General transcription factor IIF subunit 1 (Fragment) OS=Fukomys damarensis OX=885580 GN=H920_09738 PE=4 SV=1	tr|A0A091E162|A0A091E162_FUKDA (+1)	H920_09738	58 kDa	99% (1)

tRNA:

sp|Q5U2R4|TM10C_RAT tRNA methyltransferase 10 homolog C OS=Rattus norvegicus OX=10116 GN=Trmt10c PE=2 SV=1	sp|Q5U2R4|TM10C_RAT	Trmt10c	48 kDa	99% (1)
sp|Q6P7B0|SYWC_RAT Tryptophan--tRNA ligase, cytoplasmic OS=Rattus norvegicus OX=10116 GN=Wars PE=1 SV=2	sp|Q6P7B0|SYWC_RAT (+2)	Wars	54 kDa	99% (1)
tr|A0A091CTZ7|A0A091CTZ7_FUKDA Valyl-tRNA synthetase OS=Fukomys damarensis OX=885580 GN=H920_17559 PE=3 SV=1	tr|A0A091CTZ7|A0A091CTZ7_FUKDA	H920_17559	279 kDa	99% (1)
tr|A0A091D935|A0A091D935_FUKDA Putative histidyl-tRNA synthetase, mitochondrial OS=Fukomys damarensis OX=885580 GN=H920_19277 PE=4 SV=1	tr|A0A091D935|A0A091D935_FUKDA	H920_19277	57 kDa	99% (1)
tr|A0A091DKK1|A0A091DKK1_FUKDA Putative tRNA (Adenine-N(1)-)-methyltransferase catalytic subunit TRMT61B OS=Fukomys damarensis OX=885580 GN=H920_15222 PE=4 SV=1	tr|A0A091DKK1|A0A091DKK1_FUKDA	H920_15222	53 kDa	99% (1)
tr|A0A091EFK3|A0A091EFK3_FUKDA rRNA/tRNA 2'-O-methyltransferase fibrillarin-like protein 1 OS=Fukomys damarensis OX=885580 GN=H920_04477 PE=3 SV=1	tr|A0A091EFK3|A0A091EFK3_FUKDA (+2)	H920_04477	29 kDa	99% (1)
tr|A0A091EHA5|A0A091EHA5_FUKDA tRNA (Uracil-5-)-methyltransferase like protein A OS=Fukomys damarensis OX=885580 GN=H920_03849 PE=3 SV=1	tr|A0A091EHA5|A0A091EHA5_FUKDA (+2)	H920_03849	69 kDa	99% (1)
tr|A0A0P6K236|A0A0P6K236_HETGA Queuine tRNA-ribosyltransferase accessory subunit 2 OS=Heterocephalus glaber OX=10181 GN=QTRTD1 PE=3 SV=1	tr|A0A0P6K236|A0A0P6K236_HETGA (+2)	QTRTD1	40 kDa	99% (1)
tr|A0A1S3ENC1|A0A1S3ENC1_DIPOR tRNA-splicing endonuclease subunit Sen2 OS=Dipodomys ordii OX=10020 GN=Tsen2 PE=3 SV=1	tr|A0A1S3ENC1|A0A1S3ENC1_DIPOR	Tsen2	50 kDa	99% (1)
tr|A0A1S3F901|A0A1S3F901_DIPOR probable tRNA pseudouridine synthase 1 isoform X2 OS=Dipodomys ordii OX=10020 GN=Trub1 PE=3 SV=1	tr|A0A1S3F901|A0A1S3F901_DIPOR	Trub1	37 kDa	99% (1)
tr|A0A1S3FQU3|A0A1S3FQU3_DIPOR probable cysteine--tRNA ligase, mitochondrial OS=Dipodomys ordii OX=10020 GN=Cars2 PE=3 SV=1	tr|A0A1S3FQU3|A0A1S3FQU3_DIPOR	Cars2	62 kDa	99% (1)
tr|A0A1S3FRN5|A0A1S3FRN5_DIPOR tRNA methyltransferase 10 homolog A OS=Dipodomys ordii OX=10020 GN=Trmt10a PE=4 SV=1	tr|A0A1S3FRN5|A0A1S3FRN5_DIPOR	Trmt10a	39 kDa	99% (1)
tr|A0A1S3FSX0|A0A1S3FSX0_DIPOR probable tRNA methyltransferase 9-like protein OS=Dipodomys ordii OX=10020 GN=Kiaa1456 PE=4 SV=1	tr|A0A1S3FSX0|A0A1S3FSX0_DIPOR	Kiaa1456	50 kDa	99% (1)
tr|F1M9C9|F1M9C9_RAT Histidyl-tRNA synthetase 2, mitochondrial OS=Rattus norvegicus OX=10116 GN=Hars2 PE=1 SV=1	tr|F1M9C9|F1M9C9_RAT	Hars2	57 kDa	99% (1)
tr|M0R4L6|M0R4L6_RAT Glutamyl-tRNA(Gln) amidotransferase subunit B, mitochondrial OS=Rattus norvegicus OX=10116 GN=Gatb PE=1 SV=1	tr|M0R4L6|M0R4L6_RAT	Gatb	62 kDa	99% (1)
tr|Q5XI86|Q5XI86_RAT Peptidyl-tRNA hydrolase 2 OS=Rattus norvegicus OX=10116 GN=Ptrh2 PE=1 SV=1	tr|Q5XI86|Q5XI86_RAT	Ptrh2	20 kDa	99% (1)

RNA polymerase:

sp|Q68FQ7|RPAP3_RAT RNA polymerase II-associated protein 3 OS=Rattus norvegicus OX=10116 GN=Rpap3 PE=1 SV=1	sp|Q68FQ7|RPAP3_RAT	Rpap3	75 kDa	100% (2)
sp|Q561Q8|MED4_RAT Mediator of RNA polymerase II transcription subunit 4 OS=Rattus norvegicus OX=10116 GN=Med4 PE=2 SV=1	sp|Q561Q8|MED4_RAT (+3)	Med4	30 kDa	99% (1)
tr|A0A091CM74|A0A091CM74_FUKDA DNA-directed RNA polymerase I subunit RPA34 OS=Fukomys damarensis OX=885580 GN=H920_19646 PE=4 SV=1	tr|A0A091CM74|A0A091CM74_FUKDA	H920_19646	54 kDa	99% (1)
tr|A0A091CYB9|A0A091CYB9_FUKDA Mediator of RNA polymerase II transcription subunit 11 OS=Fukomys damarensis OX=885580 GN=MED11 PE=3 SV=1	tr|A0A091CYB9|A0A091CYB9_FUKDA	MED11	13 kDa	99% (1)
tr|A0A091DKK2|A0A091DKK2_FUKDA DNA-directed RNA polymerase subunit OS=Fukomys damarensis OX=885580 GN=H920_05967 PE=3 SV=1	tr|A0A091DKK2|A0A091DKK2_FUKDA	H920_05967	194 kDa	99% (1)
tr|A0A091DT96|A0A091DT96_FUKDA DNA-directed RNA polymerase III subunit RPC5 OS=Fukomys damarensis OX=885580 GN=H920_05042 PE=4 SV=1	tr|A0A091DT96|A0A091DT96_FUKDA	H920_05042	83 kDa	99% (1)
tr|A0A0S3NTA3|A0A0S3NTA3_9MURI RRN3 RNA polymerase I transcription factor homolog OS=Tokudaia muenninki OX=742503 GN=RRN3 PE=4 SV=1	tr|A0A0S3NTA3|A0A0S3NTA3_9MURI (+1)	RRN3	75 kDa	99% (1)
tr|A0A1S3GHX3|A0A1S3GHX3_DIPOR DNA-directed RNA polymerases I, II, and III subunit RPABC3 isoform X2 OS=Dipodomys ordii OX=10020 GN=Polr2h PE=4 SV=1	tr|A0A1S3GHX3|A0A1S3GHX3_DIPOR	Polr2h	14 kDa	99% (1)
tr|D3ZYB6|D3ZYB6_RAT DNA-directed RNA polymerase OS=Rattus norvegicus OX=10116 GN=Polrmt PE=3 SV=2	tr|D3ZYB6|D3ZYB6_RAT	Polrmt	136 kDa	99% (1)

Heat shock proteins:

sp|O88600|HSP74_RAT Heat shock 70 kDa protein 4 OS=Rattus norvegicus OX=10116 GN=Hspa4 PE=1 SV=1	sp|O88600|HSP74_RAT (+1)	Hspa4	94 kDa	99% (1)
tr|A0A091DFA4|A0A091DFA4_FUKDA Heat shock 70 kDa protein 12A (Fragment) OS=Fukomys damarensis OX=885580 GN=H920_09610 PE=4 SV=1	tr|A0A091DFA4|A0A091DFA4_FUKDA (+2)	H920_09610	74 kDa	99% (1)
tr|A0A0P6IYL2|A0A0P6IYL2_HETGA Heat shock factor-binding protein 1 OS=Heterocephalus glaber OX=10181 GN=HSBP1 PE=4 SV=1	tr|A0A0P6IYL2|A0A0P6IYL2_HETGA (+1)	HSBP1	9 kDa	99% (1)
tr|A0A0P6JD03|A0A0P6JD03_HETGA Heat shock 70 kDa protein 1A/1B OS=Heterocephalus glaber OX=10181 GN=HSPA1A PE=3 SV=1	tr|A0A0P6JD03|A0A0P6JD03_HETGA	HSPA1A	70 kDa	99% (1)
tr|G5BWP2|G5BWP2_HETGA Heat shock cognate 71 kDa protein OS=Heterocephalus glaber OX=10181 GN=GW7_16362 PE=4 SV=1	tr|G5BWP2|G5BWP2_HETGA	GW7_16362	14 kDa	99% (1)

Stress proteins:

tr|A0A091DZQ9|A0A091DZQ9_FUKDA Oxidative stress-induced growth inhibitor 1 OS=Fukomys damarensis OX=885580 GN=H920_02833 PE=4 SV=1	tr|A0A091DZQ9|A0A091DZQ9_FUKDA	H920_02833	92 kDa	99% (1)
tr|G5BFX7|G5BFX7_HETGA Oxidative stress-induced growth inhibitor 1 OS=Heterocephalus glaber OX=10181 GN=OSGIN1 PE=4 SV=1	tr|G5BFX7|G5BFX7_HETGA	OSGIN1	51 kDa	99% (1)

Translation initiation:

tr|F1LN59|F1LN59_RAT Eukaryotic translation initiation factor 4, gamma 2 OS=Rattus norvegicus OX=10116 GN=Eif4g2 PE=1 SV=2	tr|F1LN59|F1LN59_RAT	Eif4g2	102 kDa	100% (2)
sp|Q62818|EI2BB_RAT Translation initiation factor eIF-2B subunit beta OS=Rattus norvegicus OX=10116 GN=Eif2b2 PE=2 SV=1	sp|Q62818|EI2BB_RAT	Eif2b2	39 kDa	99% (1)
sp|Q64270|EI2BA_RAT Translation initiation factor eIF-2B subunit alpha OS=Rattus norvegicus OX=10116 GN=Eif2b1 PE=2 SV=1	sp|Q64270|EI2BA_RAT (+3)	Eif2b1	34 kDa	99% (1)
tr|A0A091DM45|A0A091DM45_FUKDA Eukaryotic translation initiation factor 4 gamma 1 OS=Fukomys damarensis OX=885580 GN=H920_07067 PE=4 SV=1	tr|A0A091DM45|A0A091DM45_FUKDA	H920_07067	20 kDa	99% (1)
tr|A0A0P6J8P2|A0A0P6J8P2_HETGA Translation initiation factor eIF-2B subunit delta isoform 3 OS=Heterocephalus glaber OX=10181 GN=EIF2B4 PE=3 SV=1	tr|A0A0P6J8P2|A0A0P6J8P2_HETGA (+1)	EIF2B4	58 kDa	99% (1)
tr|D3ZUV3|D3ZUV3_RAT Eukaryotic translation initiation factor 2A OS=Rattus norvegicus OX=10116 GN=Eif2a PE=1 SV=3	tr|D3ZUV3|D3ZUV3_RAT	Eif2a	65 kDa	99% (1)
tr|G5BAU6|G5BAU6_HETGA Eukaryotic translation initiation factor 2 subunit 2 OS=Heterocephalus glaber OX=10181 GN=GW7_19087 PE=4 SV=1	tr|G5BAU6|G5BAU6_HETGA	GW7_19087	23 kDa	99% (1)
tr|G5C5F9|G5C5F9_HETGA Eukaryotic translation initiation factor 1A OS=Heterocephalus glaber OX=10181 GN=GW7_10528 PE=4 SV=1	tr|G5C5F9|G5C5F9_HETGA	GW7_10528	7 kDa	99% (1)
tr|G5C9S0|G5C9S0_HETGA Eukaryotic translation initiation factor 2 subunit 2 OS=Heterocephalus glaber OX=10181 GN=GW7_19390 PE=4 SV=1	tr|G5C9S0|G5C9S0_HETGA	GW7_19390	26 kDa	99% (1)

Calcium:

sp|O70150-2|KCC1B_RAT Isoform 2 of Calcium/calmodulin-dependent protein kinase type 1B OS=Rattus norvegicus OX=10116 GN=Pnck	sp|O70150-2|KCC1B_RAT (+2)	Pnck	38 kDa	99% (1)
sp|O88751|CABP1_RAT Calcium-binding protein 1 OS=Rattus norvegicus OX=10116 GN=Cabp1 PE=1 SV=2	sp|O88751|CABP1_RAT	Cabp1	33 kDa	99% (1)

sp|O88831-2|KKCC2_RAT Isoform 2 of Calcium/calmodulin-dependent protein kinase kinase 2 OS=Rattus norvegicus OX=10116 GN=Camkk2	sp|O88831-2|KKCC2_RAT (+11)	Camkk2	60 kDa	99% (1)
sp|P11507-2|AT2A2_RAT Isoform 2 of Sarcoplasmic/endoplasmic reticulum calcium ATPase 2 OS=Rattus norvegicus OX=10116 GN=Atp2a2	sp|P11507-2|AT2A2_RAT (+1)	Atp2a2	110 kDa	99% (1)
sp|P70606|KCNN1_RAT Small conductance calcium-activated potassium channel protein 1 OS=Rattus norvegicus OX=10116 GN=Kcnn1 PE=1 SV=2	sp|P70606|KCNN1_RAT (+1)	Kcnn1	59 kDa	99% (1)
sp|Q66HC0|EFCB3_RAT EF-hand calcium-binding domain-containing protein 3 OS=Rattus norvegicus OX=10116 GN=Efcab3 PE=1 SV=1	sp|Q66HC0|EFCB3_RAT (+1)	Efcab3	50 kDa	99% (1)
sp|Q8R4C1|AT2C2_RAT Calcium-transporting ATPase type 2C member 2 OS=Rattus norvegicus OX=10116 GN=Atp2c2 PE=2 SV=1	sp|Q8R4C1|AT2C2_RAT (+2)	Atp2c2	103 kDa	99% (1)
tr|A0A091CWE7|A0A091CWE7_FUKDA Calcium-binding mitochondrial carrier protein Aralar1 OS=Fukomys damarensis OX=885580 GN=H920_16194 PE=3 SV=1	tr|A0A091CWE7|A0A091CWE7_FUKDA (+1)	H920_16194	75 kDa	99% (1)
tr|A0A091DQX2|A0A091DQX2_FUKDA Calcium/calmodulin-dependent protein kinase type 1D OS=Fukomys damarensis OX=885580 GN=H920_04046 PE=4 SV=1	tr|A0A091DQX2|A0A091DQX2_FUKDA	H920_04046	40 kDa	99% (1)
tr|A0A091E2H6|A0A091E2H6_FUKDA Voltage-dependent calcium channel subunit alpha-2/delta-1 (Fragment) OS=Fukomys damarensis OX=885580 GN=H920_01037 PE=4 SV=1	tr|A0A091E2H6|A0A091E2H6_FUKDA	H920_01037	106 kDa	99% (1)
tr|A0A091E627|A0A091E627_FUKDA EF-hand calcium-binding domain-containing protein 1 OS=Fukomys damarensis OX=885580 GN=H920_07944 PE=4 SV=1	tr|A0A091E627|A0A091E627_FUKDA	H920_07944	40 kDa	99% (1)
tr|A0A091EH55|A0A091EH55_FUKDA Voltage-dependent T-type calcium channel subunit alpha-1H OS=Fukomys damarensis OX=885580 GN=H920_03779 PE=4 SV=1	tr|A0A091EH55|A0A091EH55_FUKDA	H920_03779	250 kDa	99% (1)
tr|A0A1S3EPA1|A0A1S3EPA1_DIPOR EF-hand calcium-binding domain-containing protein 12 OS=Dipodomys ordii OX=10020 GN=Efcab12 PE=4 SV=1	tr|A0A1S3EPA1|A0A1S3EPA1_DIPOR	Efcab12	69 kDa	99% (1)
tr|A0A1S3EXH2|A0A1S3EXH2_DIPOR calcium/calmodulin-dependent protein kinase kinase 1 OS=Dipodomys ordii OX=10020 GN=Camkk1 PE=4 SV=1	tr|A0A1S3EXH2|A0A1S3EXH2_DIPOR	Camkk1	50 kDa	99% (1)
tr|A0A1S3GF99|A0A1S3GF99_DIPOR EF-hand calcium-binding domain-containing protein 3-like OS=Dipodomys ordii OX=10020 GN=LOC105997496 PE=4 SV=1	tr|A0A1S3GF99|A0A1S3GF99_DIPOR	LOC105997496	43 kDa	99% (1)
tr|A0A1S3GL91|A0A1S3GL91_DIPOR voltage-dependent P/Q-type calcium channel subunit alpha-1A OS=Dipodomys ordii OX=10020 GN=Cacna1a PE=3 SV=1	tr|A0A1S3GL91|A0A1S3GL91_DIPOR	Cacna1a	227 kDa	99% (1)
tr|D3ZD36|D3ZD36_RAT Ciliary-associated calcium-binding coiled-coil 1 OS=Rattus norvegicus OX=10116 GN=Cabcoco1 PE=4 SV=1	tr|D3ZD36|D3ZD36_RAT	Cabcoco1	35 kDa	99% (1)
tr|G5APS6|G5APS6_HETGA Two pore calcium channel protein 2 OS=Heterocephalus glaber OX=10181 GN=GW7_03273 PE=4 SV=1	tr|G5APS6|G5APS6_HETGA	GW7_03273	71 kDa	99% (1)
tr|G5BQM0|G5BQM0_HETGA Sodium/calcium exchanger 3 OS=Heterocephalus glaber OX=10181 GN=GW7_02730 PE=3 SV=1	tr|G5BQM0|G5BQM0_HETGA	GW7_02730	66 kDa	99% (1)
tr|G5BXU0|G5BXU0_HETGA Voltage-dependent T-type calcium channel subunit alpha-1H OS=Heterocephalus glaber OX=10181 GN=GW7_12957 PE=4 SV=1	tr|G5BXU0|G5BXU0_HETGA	GW7_12957	248 kDa	99% (1)
