# Supplementary material for: Activation of Gαq sequesters specific transcripts into Ago2 particles
Source: Sci Rep. 2022 May 24;12:8758. doi: 10.1038/s41598-022-12737-w (PMC9130320; doi:10.1038/s41598-022-12737-w)
Supplement: Supplementary file 3 — Supplementary Information 3. [file 41598_2022_12737_MOESM3_ESM.pdf]

SI Table 2 Proteins bound to Ago2 in cells stimulated with carbachol

CARBACHOL SPECIFIC

mRNA:

tr|A0A091EHJ4|A0A091EHJ4_FUKDA Insulin-like growth factor 2 mRNA-binding protein 1 OS=Fukomys damarensis OX=885580 GN=H920_03643 PE=4 SV=1	tr|A0A091EHJ4|A0A091EHJ4_FUKDA	H920_03643	18 kDa	99% (1)
tr|G5B5T6|G5B5T6_HETGA Pre-mRNA-splicing factor RBM22 (Fragment) OS=Heterocephalus glaber OX=10181 GN=GW7_21393 PE=4 SV=1	tr|G5B5T6|G5B5T6_HETGA	GW7_21393	27 kDa	99% (1)

Transcription factor:

sp|P54844|MAF_RAT Transcription factor Maf OS=Rattus norvegicus OX=10116 GN=Maf PE=1 SV=1	sp|P54844|MAF_RAT (+2)	Maf	38 kDa	99% (1)
sp|Q03062|HES5_RAT Transcription factor HES-5 OS=Rattus norvegicus OX=10116 GN=Hes5 PE=2 SV=1	sp|Q03062|HES5_RAT	Hes5	18 kDa	99% (1)
sp|Q4V8D6|BRF2_RAT Transcription factor IIIB 50 kDa subunit OS=Rattus norvegicus OX=10116 GN=Brf2 PE=2 SV=1	sp|Q4V8D6|BRF2_RAT	Brf2	47 kDa	99% (1)
sp|Q64305|PTF1A_RAT Pancreas transcription factor 1 subunit alpha OS=Rattus norvegicus OX=10116 GN=Ptf1a PE=1 SV=1	sp|Q64305|PTF1A_RAT (+1)	Ptf1a	35 kDa	99% (1)
tr|A0A091CPJ8|A0A091CPJ8_FUKDA Activating transcription factor 7-interacting protein 2 OS=Fukomys damarensis OX=885580 GN=H920_17592 PE=4 SV=1	tr|A0A091CPJ8|A0A091CPJ8_FUKDA	H920_17592	66 kDa	99% (1)
tr|A0A091D1I2|A0A091D1I2_FUKDA T-box transcription factor TBX22 OS=Fukomys damarensis OX=885580 GN=H920_12638 PE=4 SV=1	tr|A0A091D1I2|A0A091D1I2_FUKDA	H920_12638	59 kDa	99% (1)
tr|A0A0G2K2A4|A0A0G2K2A4_RAT MYB proto-oncogene, transcription factor OS=Rattus norvegicus OX=10116 GN=Myb PE=1 SV=1	tr|A0A0G2K2A4|A0A0G2K2A4_RAT	Myb	83 kDa	99% (1)
tr|A0A0P6J2L2|A0A0P6J2L2_HETGA Cyclic AMP-dependent transcription factor ATF-6 beta isoform b OS=Heterocephalus glaber OX=10181 GN=ATF6B PE=4 SV=1	tr|A0A0P6J2L2|A0A0P6J2L2_HETGA (+1)	ATF6B	76 kDa	99% (1)
tr|A0A1S3FD15|A0A1S3FD15_DIPOR doublesex- and mab-3-related transcription factor 2 OS=Dipodomys ordii OX=10020 GN=Dmrt2 PE=4 SV=1	tr|A0A1S3FD15|A0A1S3FD15_DIPOR	Dmrt2	49 kDa	99% (1)
tr|A0A1S3GBE2|A0A1S3GBE2_DIPOR cyclic AMP-dependent transcription factor ATF-6 alpha isoform X2 OS=Dipodomys ordii OX=10020 GN=Atf6 PE=4 SV=1	tr|A0A1S3GBE2|A0A1S3GBE2_DIPOR (+1)	Atf6	73 kDa	99% (1)
tr|A0A1S3GCS6|A0A1S3GCS6_DIPOR myelin transcription factor 1 OS=Dipodomys ordii OX=10020 GN=Myt1 PE=4 SV=1	tr|A0A1S3GCS6|A0A1S3GCS6_DIPOR (+1)	Myt1	129 kDa	99% (1)
tr|A0A1S3GHX6|A0A1S3GHX6_DIPOR T-box transcription factor TBX18 OS=Dipodomys ordii OX=10020 GN=Tbx18 PE=4 SV=1	tr|A0A1S3GHX6|A0A1S3GHX6_DIPOR	Tbx18	66 kDa	99% (1)
tr|F1LMD5|F1LMD5_RAT Metal response element-binding transcription factor 2 OS=Rattus norvegicus OX=10116 GN=Mtf2 PE=4 SV=1	tr|F1LMD5|F1LMD5_RAT (+1)	Mtf2	67 kDa	99% (1)
tr|F1LWK1|F1LWK1_RAT Similar to Forkhead box protein L1 (Forkhead-related protein FKHL11) (Forkhead-related transcription factor 7) (FREAC-7) OS=Rattus norvegicus OX=10116 GN=LOC680273 PE=4 SV=2	tr|F1LWK1|F1LWK1_RAT	LOC680273	25 kDa	99% (1)
tr|G5C823|G5C823_HETGA ETS-related transcription factor Elf-3 OS=Heterocephalus glaber OX=10181 GN=GW7_12034 PE=3 SV=1	tr|G5C823|G5C823_HETGA	GW7_12034	22 kDa	99% (1)
tr|G5C844|G5C844_HETGA Transcription factor BTF3 OS=Heterocephalus glaber OX=10181 GN=GW7_19899 PE=3 SV=1	tr|G5C844|G5C844_HETGA	GW7_19899	12 kDa	99% (1)
tr|Q9Z2J8|Q9Z2J8_RAT Cbfa1/Osf2 transcription factor (Fragment) OS=Rattus norvegicus OX=10116 PE=2 SV=1	tr|Q9Z2J8|Q9Z2J8_RAT		11 kDa	99% (1)

tRNA:

sp|Q3KRC5|DUS3L_RAT tRNA-dihydrouridine(47) synthase [NAD(P)(+)]-like OS=Rattus norvegicus OX=10116 GN=Dus3l PE=2 SV=1	sp|Q3KRC5|DUS3L_RAT (+1)	Dus3l	72 kDa	99% (1)
tr|A0A091DP02|A0A091DP02_FUKDA Methionyl-tRNA synthetase, cytoplasmic OS=Fukomys damarensis OX=885580 GN=H920_14022 PE=3 SV=1	tr|A0A091DP02|A0A091DP02_FUKDA (+2)	H920_14022	88 kDa	99% (1)
tr|A0A0P6JH70|A0A0P6JH70_HETGA Bifunctional glutamate/proline--tRNA ligase OS=Heterocephalus glaber OX=10181 GN=EPRS PE=3 SV=1	tr|A0A0P6JH70|A0A0P6JH70_HETGA	EPRS	169 kDa	99% (1)
tr|A0A1S3GPK5|A0A1S3GPK5_DIPOR glycine--tRNA ligase OS=Dipodomys ordii OX=10020 GN=Gars PE=4 SV=1	tr|A0A1S3GPK5|A0A1S3GPK5_DIPOR	Gars	77 kDa	99% (1)
tr|A0A1W2Q6K7|A0A1W2Q6K7_RAT Glutamyl-tRNA amidotransferase subunit B (Fragment) OS=Rattus norvegicus OX=10116 GN=Gatb PE=4 SV=1	tr|A0A1W2Q6K7|A0A1W2Q6K7_RAT	Gatb	15 kDa	99% (1)
tr|G5BMK6|G5BMK6_HETGA Asparaginyl-tRNA synthetase, cytoplasmic OS=Heterocephalus glaber OX=10181 GN=GW7_21599 PE=4 SV=1	tr|G5BMK6|G5BMK6_HETGA	GW7_21599	14 kDa	99% (1)
tr|G5C4S7|G5C4S7_HETGA tRNA (Guanine(10)-N2)-methyltransferase homolog OS=Heterocephalus glaber OX=10181 GN=TRMT11 PE=4 SV=1	tr|G5C4S7|G5C4S7_HETGA	TRMT11	53 kDa	99% (1)

RNA polymerase:

tr|A0A091CPW9|A0A091CPW9_FUKDA TAF5-like RNA polymerase II p300/CBP-associated factor-associated factor 65 kDa subunit 5L OS=Fukomys damarensis OX=885580 GN=H920_19320 PE=4 SV=1	tr|A0A091CPW9|A0A091CPW9_FUKDA	H920_19320	60 kDa	99% (1)
tr|A0A091DTW8|A0A091DTW8_FUKDA TAF5-like RNA polymerase II p300/CBP-associated factor-associated factor 65 kDa subunit 5L OS=Fukomys damarensis OX=885580 GN=H920_02996 PE=4 SV=1	tr|A0A091DTW8|A0A091DTW8_FUKDA	H920_02996	19 kDa	99% (1)
tr|A0A1S3EXC1|A0A1S3EXC1_DIPOR probable RNA polymerase II nuclear localization protein SLC7A6OS OS=Dipodomys ordii OX=10020 GN=Slc7a6os PE=4 SV=1	tr|A0A1S3EXC1|A0A1S3EXC1_DIPOR	Slc7a6os	35 kDa	99% (1)
tr|A0A1S3EZW5|A0A1S3EZW5_DIPOR TATA box-binding protein-associated factor RNA polymerase I subunit B OS=Dipodomys ordii OX=10020 GN=Taf1b PE=4 SV=1	tr|A0A1S3EZW5|A0A1S3EZW5_DIPOR	Taf1b	62 kDa	99% (1)
tr|A0A1S3F1X3|A0A1S3F1X3_DIPOR poly(A) RNA polymerase, mitochondrial isoform X2 OS=Dipodomys ordii OX=10020 GN=Mtpap PE=4 SV=1	tr|A0A1S3F1X3|A0A1S3F1X3_DIPOR (+1)	Mtpap	57 kDa	99% (1)
tr|A0A1S3GGJ3|A0A1S3GGJ3_DIPOR mediator of RNA polymerase II transcription subunit 12 OS=Dipodomys ordii OX=10020 GN=Med12 PE=4 SV=1	tr|A0A1S3GGJ3|A0A1S3GGJ3_DIPOR	Med12	244 kDa	99% (1)

Heat shock proteins:

tr|A0A0G2K435|A0A0G2K435_RAT DnaJ heat shock protein family (Hsp40) member C7 OS=Rattus norvegicus OX=10116 GN=Dnajc7 PE=1 SV=1	tr|A0A0G2K435|A0A0G2K435_RAT	Dnajc7	66 kDa	99% (1)
tr|A0A0P6J3P1|A0A0P6J3P1_HETGA Activator of 90 kDa heat shock protein ATPase homolog 2 OS=Heterocephalus glaber OX=10181 GN=AHSA2 PE=4 SV=1	tr|A0A0P6J3P1|A0A0P6J3P1_HETGA	AHSA2	18 kDa	99% (1)

Translation initiation:

sp|Q3T1J1|IF5A1_RAT Eukaryotic translation initiation factor 5A-1 OS=Rattus norvegicus OX=10116 GN=Eif5a PE=1 SV=3	sp|Q3T1J1|IF5A1_RAT (+2)	Eif5a	17 kDa	99% (1)
sp|Q5PPG7|EIF2D_RAT Eukaryotic translation initiation factor 2D OS=Rattus norvegicus OX=10116 GN=Eif2d PE=1 SV=1	sp|Q5PPG7|EIF2D_RAT	Eif2d	63 kDa	99% (1)
sp|Q64350|EI2BE_RAT Translation initiation factor eIF-2B subunit epsilon OS=Rattus norvegicus OX=10116 GN=Eif2b5 PE=1 SV=2	sp|Q64350|EI2BE_RAT	Eif2b5	80 kDa	99% (1)
tr|A0A0G2K273|A0A0G2K273_RAT Eukaryotic translation initiation factor 3 subunit E OS=Rattus norvegicus OX=10116 GN=LOC100909481 PE=3 SV=1	tr|A0A0G2K273|A0A0G2K273_RAT	LOC100909481	52 kDa	99% (1)
sp|Q3B8Q2|IF4A3_RAT Eukaryotic initiation factor 4A-III OS=Rattus norvegicus OX=10116 GN=Eif4a3 PE=1 SV=1	sp|Q3B8Q2|IF4A3_RAT	Eif4a3	47 kDa	99% (1)
tr|A0A0P6K2W0|A0A0P6K2W0_HETGA Interferon-induced, double-stranded RNA-activated protein kinase isoform a OS=Heterocephalus glaber OX=10181 GN=EIF2AK2 PE=4 SV=1	tr|A0A0P6K2W0|A0A0P6K2W0_HETGA (+1)	EIF2AK2	61 kDa	99% (1)

Calcium:

sp|A7VL23|KCMB3_RAT Calcium-activated potassium channel subunit beta-3 OS=Rattus norvegicus OX=10116 GN=Kcnmb3 PE=2 SV=1	sp|A7VL23|KCMB3_RAT	Kcnmb3	26 kDa	99% (1)
sp|P70549|NAC3_RAT Sodium/calcium exchanger 3 OS=Rattus norvegicus OX=10116 GN=Slc8a3 PE=1 SV=1	sp|P70549|NAC3_RAT (+1)	Slc8a3	103 kDa	99% (1)
sp|Q63421|PDE1C_RAT Calcium/calmodulin-dependent 3',5'-cyclic nucleotide phosphodiesterase 1C OS=Rattus norvegicus OX=10116 GN=Pde1c PE=1 SV=1	sp|Q63421|PDE1C_RAT (+5)	Pde1c	87 kDa	99% (1)
sp|Q7TNJ7|KCC1G_RAT Calcium/calmodulin-dependent protein kinase type 1G OS=Rattus norvegicus OX=10116 GN=Camk1g PE=2 SV=1	sp|Q7TNJ7|KCC1G_RAT	Camk1g	53 kDa	99% (1)
sp|Q9Z0Y8-2|CAC1I_RAT Isoform 2 of Voltage-dependent T-type calcium channel subunit alpha-1I OS=Rattus norvegicus OX=10116 GN=Cacna1i	sp|Q9Z0Y8-2|CAC1I_RAT (+1)	Cacna1i	205 kDa	99% (1)
tr|A0A091DKT8|A0A091DKT8_FUKDA Calcium-transporting ATPase OS=Fukomys damarensis OX=885580 GN=H920_05868 PE=3 SV=1	tr|A0A091DKT8|A0A091DKT8_FUKDA (+3)	H920_05868	107 kDa	99% (1)
tr|A0A091DPZ8|A0A091DPZ8_FUKDA Calcium homeostasis modulator protein 2 OS=Fukomys damarensis OX=885580 GN=H920_06282 PE=4 SV=1	tr|A0A091DPZ8|A0A091DPZ8_FUKDA	H920_06282	101 kDa	99% (1)
tr|A0A091DRM2|A0A091DRM2_FUKDA EF-hand calcium-binding domain-containing protein 2 OS=Fukomys damarensis OX=885580 GN=H920_13222 PE=4 SV=1	tr|A0A091DRM2|A0A091DRM2_FUKDA	H920_13222	15 kDa	99% (1)
tr|A0A091DS09|A0A091DS09_FUKDA Calcium-binding and coiled-coil domain-containing protein 2 OS=Fukomys damarensis OX=885580 GN=H920_03641 PE=3 SV=1	tr|A0A091DS09|A0A091DS09_FUKDA	H920_03641	100 kDa	99% (1)
tr|A0A091E3P3|A0A091E3P3_FUKDA Sodium/potassium/calcium exchanger 5 OS=Fukomys damarensis OX=885580 GN=H920_08826 PE=3 SV=1	tr|A0A091E3P3|A0A091E3P3_FUKDA	H920_08826	56 kDa	99% (1)
tr|A0A091ELJ3|A0A091ELJ3_FUKDA Extracellular calcium-sensing receptor OS=Fukomys damarensis OX=885580 GN=H920_02083 PE=4 SV=1	tr|A0A091ELJ3|A0A091ELJ3_FUKDA	H920_02083	75 kDa	99% (1)
tr|A0A1S3FU28|A0A1S3FU28_DIPOR calcium uptake protein 3, mitochondrial OS=Dipodomys ordii OX=10020 GN=Micu3 PE=4 SV=1	tr|A0A1S3FU28|A0A1S3FU28_DIPOR (+2)	Micu3	50 kDa	99% (1)
tr|A0A1S3GJ12|A0A1S3GJ12_DIPOR EF-hand calcium-binding domain-containing protein 13 OS=Dipodomys ordii OX=10020 GN=Efcab13 PE=4 SV=1	tr|A0A1S3GJ12|A0A1S3GJ12_DIPOR	Efcab13	243 kDa	99% (1)
tr|G5APP2|G5APP2_HETGA Tumor-associated calcium signal transducer 2 OS=Heterocephalus glaber OX=10181 GN=GW7_13500 PE=4 SV=1	tr|G5APP2|G5APP2_HETGA	GW7_13500	26 kDa	99% (1)
tr|G5AQP6|G5AQP6_HETGA Voltage-dependent calcium channel subunit alpha-2/delta-4 (Fragment) OS=Heterocephalus glaber OX=10181 GN=GW7_05647 PE=4 SV=1	tr|G5AQP6|G5AQP6_HETGA	GW7_05647	128 kDa	99% (1)
tr|G5BJB7|G5BJB7_HETGA Sodium/calcium exchanger 1 OS=Heterocephalus glaber OX=10181 GN=GW7_11585 PE=3 SV=1	tr|G5BJB7|G5BJB7_HETGA	GW7_11585	65 kDa	99% (1)
sp|P11505-1|AT2B1_RAT Isoform D of Plasma membrane calcium-transporting ATPase 1 OS=Rattus norvegicus OX=10116 GN=Atp2b1	sp|P11505-1|AT2B1_RAT	Atp2b1	139 kDa	100% (2)
tr|A0A1S3GEQ6|A0A1S3GEQ6_DIPOR calcium-binding mitochondrial carrier protein SCaMC-1-like OS=Dipodomys ordii OX=10020 GN=LOC105997461 PE=3 SV=1	tr|A0A1S3GEQ6|A0A1S3GEQ6_DIPOR	LOC105997461	54 kDa	100% (2)
