# Supplementary material for: Activation of Gαq sequesters specific transcripts into Ago2 particles
Source: Sci Rep. 2022 May 24;12:8758. doi: 10.1038/s41598-022-12737-w (PMC9130320; doi:10.1038/s41598-022-12737-w)
Supplement: Supplementary file 4 — Supplementary Information 4. [file 41598_2022_12737_MOESM4_ESM.pdf]

SI Table 3 Proteins associated with Ago2 in cells subjected to heat shock

HEAT SPECIFIC

mRNA:					
	tr|A0A091D5H0|A0A091D5H0_FUKDA Pre-mRNA-processing-splicing factor 8 OS=Fukomys damarensis OX=885580 GN=H920_13028 PE=4 SV=1	tr|A0A091D5H0|A0A091D5H0_FUKDA (+3)	H920_13028	274 kDa	99% (1)
	tr|A0A091D738|A0A091D738_FUKDA Insulin-like growth factor 2 mRNA-binding protein 3 OS=Fukomys damarensis OX=885580 GN=H920_12544 PE=4 SV=1	tr|A0A091D738|A0A091D738_FUKDA	H920_12544	59 kDa	99% (1)
	tr|A0A091DHN4|A0A091DHN4_FUKDA mRNA-decapping enzyme 1B OS=Fukomys damarensis OX=885580 GN=H920_07975 PE=4 SV=1	tr|A0A091DHN4|A0A091DHN4_FUKDA	H920_07975	65 kDa	99% (1)
	tr|A0A091DJM2|A0A091DJM2_FUKDA Pre-mRNA cleavage complex 2 protein Pcf11 OS=Fukomys damarensis OX=885580 GN=H920_08112 PE=4 SV=1	tr|A0A091DJM2|A0A091DJM2_FUKDA	H920_08112	188 kDa	99% (1)
	tr|A0A1S3EVL7|A0A1S3EVL7_DIPOR cap-specific mRNA (Nucleoside-2'-O-)-methyltransferase 2 OS=Dipodomys ordii OX=10020 GN=Cmtr2 PE=4 SV=1	tr|A0A1S3EVL7|A0A1S3EVL7_DIPOR	Cmtr2	88 kDa	99% (1)
	tr|A0A1S3EYP3|A0A1S3EYP3_DIPOR pre-mRNA cleavage complex 2 protein Pcf11 isoform X2 OS=Dipodomys ordii OX=10020 GN=Pcf11 PE=4 SV=1	tr|A0A1S3EYP3|A0A1S3EYP3_DIPOR (+2)	Pcf11	186 kDa	99% (1)
Transcription factors:					
	sp|A2VD12|PBIP1_RAT Pre-B-cell leukemia transcription factor-interacting protein 1 OS=Rattus norvegicus OX=10116 GN=Pbxip1 PE=1 SV=1	sp|A2VD12|PBIP1_RAT (+1)	Pbxip1	80 kDa	99% (1)
	sp|P56222|PO3F2_RAT POU domain, class 3, transcription factor 2 OS=Rattus norvegicus OX=10116 GN=Pou3f2 PE=1 SV=1	sp|P56222|PO3F2_RAT (+1)	Pou3f2	47 kDa	99% (1)
	sp|P56225|PO5F2_RAT POU domain, class 5, transcription factor 2 OS=Rattus norvegicus OX=10116 GN=Pou5f2 PE=2 SV=2	sp|P56225|PO5F2_RAT	Pou5f2	37 kDa	99% (1)
	sp|P70475|MYT1L_RAT Myelin transcription factor 1-like protein OS=Rattus norvegicus OX=10116 GN=Myt1l PE=1 SV=3	sp|P70475|MYT1L_RAT (+1)	Myt1l	133 kDa	99% (1)
	tr|A0A091CPH2|A0A091CPH2_FUKDA Doublesex-and mab-3-related transcription factor B1 OS=Fukomys damarensis OX=885580 GN=H920_17737 PE=4 SV=1	tr|A0A091CPH2|A0A091CPH2_FUKDA	H920_17737	28 kDa	99% (1)
	tr|A0A091CYW2|A0A091CYW2_FUKDA Transcription factor ETV6 OS=Fukomys damarensis OX=885580 GN=H920_13585 PE=3 SV=1	tr|A0A091CYW2|A0A091CYW2_FUKDA	H920_13585	63 kDa	99% (1)
	tr|A0A091E720|A0A091E720_FUKDA Cyclic AMP-dependent transcription factor ATF-6 alpha OS=Fukomys damarensis OX=885580 GN=H920_00195 PE=4 SV=1	tr|A0A091E720|A0A091E720_FUKDA	H920_00195	62 kDa	99% (1)
	tr|A0A0N8ET04|A0A0N8ET04_HETGA T-box transcription factor TBX19 OS=Heterocephalus glaber OX=10181 GN=TBX19 PE=4 SV=1	tr|A0A0N8ET04|A0A0N8ET04_HETGA (+1)	TBX19	48 kDa	99% (1)
	tr|A0A1S3EJC7|A0A1S3EJC7_DIPOR RE1-silencing transcription factor isoform X2 OS=Dipodomys ordii OX=10020 GN=Rest PE=4 SV=1	tr|A0A1S3EJC7|A0A1S3EJC7_DIPOR (+1)	Rest	117 kDa	99% (1)
	tr|A0A1S3ENU9|A0A1S3ENU9_DIPOR transcription factor 12 isoform X9 OS=Dipodomys ordii OX=10020 GN=Tcf12 PE=4 SV=1	tr|A0A1S3ENU9|A0A1S3ENU9_DIPOR	Tcf12	47 kDa	99% (1)
	tr|A0A1S3FBE3|A0A1S3FBE3_DIPOR doublesex- and mab-3-related transcription factor 1 OS=Dipodomys ordii OX=10020 GN=Dmrt1 PE=4 SV=1	tr|A0A1S3FBE3|A0A1S3FBE3_DIPOR (+1)	Dmrt1	28 kDa	99% (1)
	tr|A0A1S3FJB4|A0A1S3FJB4_DIPOR transcription factor CP2-like protein 1 OS=Dipodomys ordii OX=10020 GN=Tfcp2l1 PE=4 SV=1	tr|A0A1S3FJB4|A0A1S3FJB4_DIPOR (+1)	Tfcp2l1	55 kDa	99% (1)
	tr|A0A1S3FX19|A0A1S3FX19_DIPOR LOW QUALITY PROTEIN: transcription factor SOX-10 OS=Dipodomys ordii OX=10020 GN=Sox10 PE=4 SV=1	tr|A0A1S3FX19|A0A1S3FX19_DIPOR	Sox10	50 kDa	99% (1)
	tr|D3ZLV5|D3ZLV5_RAT AT-hook transcription factor OS=Rattus norvegicus OX=10116 GN=Akna PE=1 SV=1	tr|D3ZLV5|D3ZLV5_RAT	Akna	153 kDa	99% (1)
	tr|D3ZZ13|D3ZZ13_RAT Transcription factor SOX OS=Rattus norvegicus OX=10116 GN=Sox12 PE=4 SV=1	tr|D3ZZ13|D3ZZ13_RAT	Sox12	34 kDa	99% (1)
	tr|G5AM15|G5AM15_HETGA Runt-related transcription factor OS=Heterocephalus glaber OX=10181 GN=GW7_16276 PE=4 SV=1	tr|G5AM15|G5AM15_HETGA	GW7_16276	54 kDa	99% (1)
	tr|G5C3D4|G5C3D4_HETGA Transcription factor 19 OS=Heterocephalus glaber OX=10181 GN=GW7_04764 PE=4 SV=1	tr|G5C3D4|G5C3D4_HETGA	GW7_04764	37 kDa	99% (1)
	tr|Q4Z8P1|Q4Z8P1_RAT Fli-1 proto-oncogene, ETS transcription factor OS=Rattus norvegicus OX=10116 GN=Fli1 PE=1 SV=1	tr|Q4Z8P1|Q4Z8P1_RAT	Fli1	51 kDa	99% (1)
tRNA:					
	sp|Q68FW7|SYTM_RAT Threonine--tRNA ligase, mitochondrial OS=Rattus norvegicus OX=10116 GN=Tars2 PE=2 SV=1	sp|Q68FW7|SYTM_RAT	Tars2	82 kDa	99% (1)
	tr|A0A0N8EU10|A0A0N8EU10_HETGA tRNA wybutosine-synthesizing protein 3 homolog isoform 2 OS=Heterocephalus glaber OX=10181 GN=TYW3 PE=4 SV=1	tr|A0A0N8EU10|A0A0N8EU10_HETGA	TYW3	48 kDa	99% (1)
	tr|A0A1S3GPK8|A0A1S3GPK8_DIPOR tRNA-dihydrouridine synthase OS=Dipodomys ordii OX=10020 GN=Dus4l PE=3 SV=1	tr|A0A1S3GPK8|A0A1S3GPK8_DIPOR	Dus4l	36 kDa	99% (1)
	tr|G5BH39|G5BH39_HETGA Threonyl-tRNA synthetase, mitochondrial OS=Heterocephalus glaber OX=10181 GN=GW7_05802 PE=3 SV=1	tr|G5BH39|G5BH39_HETGA	GW7_05802	81 kDa	99% (1)
	tr|G5BJ35|G5BJ35_HETGA Threonyl-tRNA synthetase, cytoplasmic OS=Heterocephalus glaber OX=10181 GN=GW7_21114 PE=3 SV=1	tr|G5BJ35|G5BJ35_HETGA	GW7_21114	92 kDa	99% (1)
	tr|G5BMJ2|G5BMJ2_HETGA Glutamyl-tRNA(Gln) amidotransferase subunit B, mitochondrial OS=Heterocephalus glaber OX=10181 GN=GATB PE=3 SV=1	tr|G5BMJ2|G5BMJ2_HETGA	GATB	69 kDa	99% (1)
	tr|G5C2Y2|G5C2Y2_HETGA Putative tRNA (Adenine(58)-N(1))-methyltransferase catalytic subunit TRMT61B OS=Heterocephalus glaber OX=10181 GN=TRMT61B PE=4 SV=1	tr|G5C2Y2|G5C2Y2_HETGA	TRMT61B	54 kDa	99% (1)
	tr|G5C686|G5C686_HETGA tRNA-dihydrouridine synthase 2-like protein OS=Heterocephalus glaber OX=10181 GN=GW7_02382 PE=4 SV=1	tr|G5C686|G5C686_HETGA	GW7_02382	55 kDa	99% (1)
RNA polymerase:					
	tr|A0A091D4E6|A0A091D4E6_FUKDA TATA box-binding protein-associated factor RNA polymerase I subunit A OS=Fukomys damarensis OX=885580 GN=H920_13144 PE=4 SV=1	tr|A0A091D4E6|A0A091D4E6_FUKDA (+2)	H920_13144	68 kDa	99% (1)
	tr|A0A091DAU9|A0A091DAU9_FUKDA Mediator of RNA polymerase II transcription subunit 17 OS=Fukomys damarensis OX=885580 GN=MED17 PE=3 SV=1	tr|A0A091DAU9|A0A091DAU9_FUKDA (+1)	MED17	73 kDa	99% (1)
	tr|A0A091EHB9|A0A091EHB9_FUKDA Mediator of RNA polymerase II transcription subunit 15 OS=Fukomys damarensis OX=885580 GN=MED15 PE=3 SV=1	tr|A0A091EHB9|A0A091EHB9_FUKDA (+2)	MED15	80 kDa	99% (1)
	tr|A0A0G2JTZ3|A0A0G2JTZ3_RAT TATA box-binding protein-associated factor RNA polymerase I subunit A OS=Rattus norvegicus OX=10116 GN=Taf1a PE=4 SV=1	tr|A0A0G2JTZ3|A0A0G2JTZ3_RAT	Taf1a	53 kDa	99% (1)
	tr|A0A0P6J8D2|A0A0P6J8D2_HETGA Mediator of RNA polymerase II transcription subunit 1 OS=Heterocephalus glaber OX=10181 GN=MED1 PE=3 SV=1	tr|A0A0P6J8D2|A0A0P6J8D2_HETGA	MED1	168 kDa	99% (1)
	tr|A0A0P6K376|A0A0P6K376_HETGA RNA polymerase I-specific transcription initiation factor RRN3 OS=Heterocephalus glaber OX=10181 GN=RRN3 PE=4 SV=1	tr|A0A0P6K376|A0A0P6K376_HETGA (+1)	RRN3	75 kDa	99% (1)
	tr|A0A1S3EPP9|A0A1S3EPP9_DIPOR RNA polymerase II-associated protein 1 OS=Dipodomys ordii OX=10020 GN=Rpap1 PE=4 SV=1	tr|A0A1S3EPP9|A0A1S3EPP9_DIPOR	Rpap1	134 kDa	99% (1)
	tr|A0A1S3FAY6|A0A1S3FAY6_DIPOR DNA-directed RNA polymerase III subunit RPC7-like isoform X2 OS=Dipodomys ordii OX=10020 GN=Polr3gl PE=4 SV=1	tr|A0A1S3FAY6|A0A1S3FAY6_DIPOR	Polr3gl	18 kDa	99% (1)
Heat shock proteins:					
	tr|G5BWG9|G5BWG9_HETGA Heat shock 70 kDa protein 6 OS=Heterocephalus glaber OX=10181 GN=GW7_00490 PE=4 SV=1	tr|G5BWG9|G5BWG9_HETGA	GW7_00490	26 kDa	99% (1)
Stress proteins:					
	tr|A0A1S3GXP3|A0A1S3GXP3_DIPOR Stress-associated endoplasmic reticulum protein OS=Dipodomys ordii OX=10020 GN=Serp2 PE=3 SV=1	tr|A0A1S3GXP3|A0A1S3GXP3_DIPOR (+2)	Serp2	7 kDa	99% (1)
Translation initiation:					
	sp|P70541|EI2BG_RAT Translation initiation factor eIF-2B subunit gamma OS=Rattus norvegicus OX=10116 GN=Eif2b3 PE=2 SV=2	sp|P70541|EI2BG_RAT	Eif2b3	50 kDa	99% (1)
	sp|Q5XI72|IF4H_RAT Eukaryotic translation initiation factor 4H OS=Rattus norvegicus OX=10116 GN=Eif4h PE=1 SV=1	sp|Q5XI72|IF4H_RAT (+6)	Eif4h	27 kDa	99% (1)
	tr|A0A1L1WKF3|A0A1L1WKF3_NINCN Eukaryotic translation initiation factor 2B (Fragment) OS=Niviventer confucianus OX=248811 PE=2 SV=1	tr|A0A1L1WKF3|A0A1L1WKF3_NINCN		22 kDa	99% (1)
	tr|G5BP48|G5BP48_HETGA Eukaryotic translation initiation factor 1A, X-chromosomal OS=Heterocephalus glaber OX=10181 GN=GW7_09468 PE=4 SV=1	tr|G5BP48|G5BP48_HETGA	GW7_09468	12 kDa	99% (1)
Calcium:					
	tr|A0A0P6K3B4|A0A0P6K3B4_HETGA 85 kDa calcium-independent phospholipase A2 isoform b OS=Heterocephalus glaber OX=10181 GN=PLA2G6 PE=4 SV=1	tr|A0A0P6K3B4|A0A0P6K3B4_HETGA (+1)	PLA2G6	84 kDa	100% (2)
	sp|P11506-10|AT2B2_RAT Isoform XC of Plasma membrane calcium-transporting ATPase 2 OS=Rattus norvegicus OX=10116 GN=Atp2b2	sp|P11506-10|AT2B2_RAT (+16)	Atp2b2	130 kDa	99% (1)
	sp|Q8VHW7-2|CCG6_RAT Isoform Short of Voltage-dependent calcium channel gamma-6 subunit OS=Rattus norvegicus OX=10116 GN=Cacng6	sp|Q8VHW7-2|CCG6_RAT (+1)	Cacng6	24 kDa	99% (1)
	sp|Q91XJ0|CREST_RAT Calcium-responsive transcription coactivator OS=Rattus norvegicus OX=10116 GN=Ss18l1 PE=1 SV=1	sp|Q91XJ0|CREST_RAT	Ss18l1	44 kDa	99% (1)
	tr|A0A091DAS0|A0A091DAS0_FUKDA Two pore calcium channel protein 2 OS=Fukomys damarensis OX=885580 GN=H920_11257 PE=4 SV=1	tr|A0A091DAS0|A0A091DAS0_FUKDA	H920_11257	103 kDa	99% (1)
	tr|A0A091E0V8|A0A091E0V8_FUKDA Calcium uptake protein 1, mitochondrial OS=Fukomys damarensis OX=885580 GN=H920_01629 PE=4 SV=1	tr|A0A091E0V8|A0A091E0V8_FUKDA	H920_01629	54 kDa	99% (1)
	tr|A0A091EN06|A0A091EN06_FUKDA Calcium-activated potassium channel subunit alpha-1 OS=Fukomys damarensis OX=885580 GN=H920_01668 PE=3 SV=1	tr|A0A091EN06|A0A091EN06_FUKDA	H920_01668	131 kDa	99% (1)
	tr|A0A1L1WKE8|A0A1L1WKE8_RAT Calcium modulating ligand (Fragment) OS=Rattus norvegicus OX=10116 PE=2 SV=1	tr|A0A1L1WKE8|A0A1L1WKE8_RAT (+1)		24 kDa	99% (1)
	tr|A0A1S3EPI6|A0A1S3EPI6_DIPOR C2 calcium-dependent domain-containing protein 4A OS=Dipodomys ordii OX=10020 GN=C2cd4a PE=4 SV=1	tr|A0A1S3EPI6|A0A1S3EPI6_DIPOR	C2cd4a	74 kDa	99% (1)
	tr|A0A1S3FBW2|A0A1S3FBW2_DIPOR sodium/calcium exchanger 1 isoform X2 OS=Dipodomys ordii OX=10020 GN=Slc8a1 PE=3 SV=1	tr|A0A1S3FBW2|A0A1S3FBW2_DIPOR	Slc8a1	104 kDa	99% (1)
	tr|A0A1S3FPF3|A0A1S3FPF3_DIPOR C2 calcium-dependent domain-containing protein 4C OS=Dipodomys ordii OX=10020 GN=C2cd4c PE=4 SV=1	tr|A0A1S3FPF3|A0A1S3FPF3_DIPOR	C2cd4c	45 kDa	99% (1)
	tr|A0A1S3FTJ2|A0A1S3FTJ2_DIPOR EF-hand calcium-binding domain-containing protein 4B OS=Dipodomys ordii OX=10020 GN=Cracr2a PE=4 SV=1	tr|A0A1S3FTJ2|A0A1S3FTJ2_DIPOR	Cracr2a	46 kDa	99% (1)
	tr|A0A1S3GMY9|A0A1S3GMY9_DIPOR EF-hand calcium-binding domain-containing protein 10-like OS=Dipodomys ordii OX=10020 GN=LOC105999654 PE=4 SV=1	tr|A0A1S3GMY9|A0A1S3GMY9_DIPOR	LOC105999654	17 kDa	99% (1)
	tr|G5BAF4|G5BAF4_HETGA Sarcoplasmic reticulum histidine-rich calcium-binding protein OS=Heterocephalus glaber OX=10181 GN=GW7_13612 PE=4 SV=1	tr|G5BAF4|G5BAF4_HETGA	GW7_13612	67 kDa	99% (1)
	tr|G5BDD6|G5BDD6_HETGA Small conductance calcium-activated potassium channel protein 3 OS=Heterocephalus glaber OX=10181 GN=GW7_05186 PE=4 SV=1	tr|G5BDD6|G5BDD6_HETGA	GW7_05186	82 kDa	99% (1)
