# Supplementary material for: Activation of Gαq sequesters specific transcripts into Ago2 particles
Source: Sci Rep. 2022 May 24;12:8758. doi: 10.1038/s41598-022-12737-w (PMC9130320; doi:10.1038/s41598-022-12737-w)
Supplement: Supplementary file 5 — Supplementary Information 5. [file 41598_2022_12737_MOESM5_ESM.pdf]

SI Table 4 Proteins associated with Ago2 in cells subjected to hypo-osmotic stress

OSMOTIC SPECIFIC

mRNA:

sp|Q80ZG5|SLU7_RAT Pre-mRNA-splicing factor SLU7 OS=Rattus norvegicus OX=10116 GN=Slu7 PE=1 SV=2	sp|Q80ZG5|SLU7_RAT (+1)	Slu7	68 kDa	99% (1)
tr|A0A091CSJ7|A0A091CSJ7_FUKDA Putative pre-mRNA-splicing factor ATP-dependent RNA helicase DHX15 OS=Fukomys damarensis OX=885580 GN=H920_17774 PE=4 SV=1	tr|A0A091CSJ7|A0A091CSJ7_FUKDA (+2)	H920_17774	91 kDa	99% (1)
tr|A0A0U1RRY8|A0A0U1RRY8_RAT UPF2, regulator of nonsense mediated mRNA decay (Fragment) OS=Rattus norvegicus OX=10116 GN=Upf2 PE=1 SV=1	tr|A0A0U1RRY8|A0A0U1RRY8_RAT (+1)	Upf2	58 kDa	99% (1)
tr|G5AZF4|G5AZF4_HETGA Pre-mRNA-splicing regulator WTAP OS=Heterocephalus glaber OX=10181 GN=GW7_06519 PE=4 SV=1	tr|G5AZF4|G5AZF4_HETGA	GW7_06519	35 kDa	99% (1)
tr|G5B7R6|G5B7R6_HETGA Pre-mRNA-processing factor 40-like protein A OS=Heterocephalus glaber OX=10181 GN=GW7_17978 PE=4 SV=1	tr|G5B7R6|G5B7R6_HETGA	GW7_17978	76 kDa	99% (1)
tr|Q9QWQ2|Q9QWQ2_RAT Rat (wistar) alpha-2u globulin mRNA (Fragment) OS=Rattus norvegicus OX=10116 PE=3 SV=1	tr|Q9QWQ2|Q9QWQ2_RAT		17 kDa	99% (1)

Transcription Factors:

sp|Q01750|T2FB_RAT General transcription factor IIF subunit 2 OS=Rattus norvegicus OX=10116 GN=Gtf2f2 PE=1 SV=1	sp|Q01750|T2FB_RAT (+4)	Gtf2f2	28 kDa	99% (1)
tr|A0A091CV02|A0A091CV02_FUKDA Transcription factor YY2 OS=Fukomys damarensis OX=885580 GN=H920_16257 PE=4 SV=1	tr|A0A091CV02|A0A091CV02_FUKDA	H920_16257	69 kDa	99% (1)
tr|A0A091CXQ7|A0A091CXQ7_FUKDA Transcription factor E3 OS=Fukomys damarensis OX=885580 GN=H920_15943 PE=4 SV=1	tr|A0A091CXQ7|A0A091CXQ7_FUKDA (+3)	H920_15943	62 kDa	99% (1)
tr|A0A091D4T4|A0A091D4T4_FUKDA Transcription factor 12 OS=Fukomys damarensis OX=885580 GN=H920_12613 PE=4 SV=1	tr|A0A091D4T4|A0A091D4T4_FUKDA (+6)	H920_12613	52 kDa	99% (1)
tr|A0A091DBZ6|A0A091DBZ6_FUKDA ETS-related transcription factor Elf-2 OS=Fukomys damarensis OX=885580 GN=H920_10879 PE=4 SV=1	tr|A0A091DBZ6|A0A091DBZ6_FUKDA	H920_10879	13 kDa	99% (1)
tr|A0A091DH54|A0A091DH54_FUKDA T-box transcription factor TBX18 OS=Fukomys damarensis OX=885580 GN=H920_08237 PE=4 SV=1	tr|A0A091DH54|A0A091DH54_FUKDA (+1)	H920_08237	49 kDa	99% (1)
tr|A0A091DPX1|A0A091DPX1_FUKDA Transcription factor LBX1 OS=Fukomys damarensis OX=885580 GN=H920_06247 PE=4 SV=1	tr|A0A091DPX1|A0A091DPX1_FUKDA (+1)	H920_06247	19 kDa	99% (1)
tr|A0A091E2P8|A0A091E2P8_FUKDA Thyroid transcription factor 1-associated protein 26 OS=Fukomys damarensis OX=885580 GN=H920_01771 PE=4 SV=1	tr|A0A091E2P8|A0A091E2P8_FUKDA	H920_01771	28 kDa	99% (1)
tr|A0A091E2Y0|A0A091E2Y0_FUKDA Transcription factor IIIB 50 kDa subunit OS=Fukomys damarensis OX=885580 GN=H920_01539 PE=4 SV=1	tr|A0A091E2Y0|A0A091E2Y0_FUKDA	H920_01539	47 kDa	99% (1)
tr|A0A1S3F625|A0A1S3F625_DIPOR transcription factor TFIIIB component B'' homolog OS=Dipodomys ordii OX=10020 GN=Bdp1 PE=4 SV=1	tr|A0A1S3F625|A0A1S3F625_DIPOR	Bdp1	228 kDa	99% (1)
tr|A0A1S3G2E8|A0A1S3G2E8_DIPOR Runt-related transcription factor OS=Dipodomys ordii OX=10020 GN=Runx1 PE=4 SV=1	tr|A0A1S3G2E8|A0A1S3G2E8_DIPOR (+1)	Runx1	50 kDa	99% (1)
tr|A0A1S3G5A1|A0A1S3G5A1_DIPOR HMG box transcription factor BBX isoform X1 OS=Dipodomys ordii OX=10020 GN=Bbx PE=4 SV=1	tr|A0A1S3G5A1|A0A1S3G5A1_DIPOR (+2)	Bbx	108 kDa	99% (1)
tr|A0A1S3GC22|A0A1S3GC22_DIPOR AT-hook-containing transcription factor OS=Dipodomys ordii OX=10020 GN=Akna PE=4 SV=1	tr|A0A1S3GC22|A0A1S3GC22_DIPOR	Akna	146 kDa	99% (1)
tr|G5AT36|G5AT36_HETGA General transcription factor 3C polypeptide 5 OS=Heterocephalus glaber OX=10181 GN=GW7_03375 PE=4 SV=1	tr|G5AT36|G5AT36_HETGA	GW7_03375	62 kDa	99% (1)
tr|G5AVV2|G5AVV2_HETGA Transcription factor Dp-1 (Fragment) OS=Heterocephalus glaber OX=10181 GN=GW7_08579 PE=3 SV=1	tr|G5AVV2|G5AVV2_HETGA	GW7_08579	44 kDa	99% (1)
tr|G5C6B2|G5C6B2_HETGA General transcription factor II-I repeat domain-containing protein 2B OS=Heterocephalus glaber OX=10181 GN=GW7_12170 PE=4 SV=1	tr|G5C6B2|G5C6B2_HETGA	GW7_12170	106 kDa	99% (1)

tRNA:

sp|Q3KRD0|SYDM_RAT Aspartate--tRNA ligase, mitochondrial OS=Rattus norvegicus OX=10116 GN=Dars2 PE=1 SV=1	sp|Q3KRD0|SYDM_RAT	Dars2	74 kDa	99% (1)
tr|A0A091CUK8|A0A091CUK8_FUKDA tRNA (adenine(58)-N(1))-methyltransferase non-catalytic subunit TRM6 OS=Fukomys damarensis OX=885580 GN=H920_16421 PE=3 SV=1	tr|A0A091CUK8|A0A091CUK8_FUKDA (+4)	H920_16421	56 kDa	99% (1)
tr|A0A091DEA6|A0A091DEA6_FUKDA tRNA-dihydrouridine(47) synthase [NAD(P)(+)] OS=Fukomys damarensis OX=885580 GN=H920_09750 PE=3 SV=1	tr|A0A091DEA6|A0A091DEA6_FUKDA	H920_09750	61 kDa	99% (1)
tr|A0A091DUF5|A0A091DUF5_FUKDA tRNA-dihydrouridine synthase 1-like OS=Fukomys damarensis OX=885580 GN=H920_02958 PE=4 SV=1	tr|A0A091DUF5|A0A091DUF5_FUKDA (+2)	H920_02958	54 kDa	99% (1)
tr|A0A091E906|A0A091E906_FUKDA Glutamyl-tRNA(Gln) amidotransferase subunit B, mitochondrial OS=Fukomys damarensis OX=885580 GN=GATB PE=3 SV=1	tr|A0A091E906|A0A091E906_FUKDA	GATB	62 kDa	99% (1)
tr|A0A091E9L2|A0A091E9L2_FUKDA Putative tRNA pseudouridine synthase 2 OS=Fukomys damarensis OX=885580 GN=H920_06647 PE=4 SV=1	tr|A0A091E9L2|A0A091E9L2_FUKDA	H920_06647	37 kDa	99% (1)
tr|A0A0P6J3Q6|A0A0P6J3Q6_HETGA tRNA (Cytosine(34)-C(5))-methyltransferase isoform 2 OS=Heterocephalus glaber OX=10181 GN=NSUN2 PE=3 SV=1	tr|A0A0P6J3Q6|A0A0P6J3Q6_HETGA (+1)	NSUN2	86 kDa	99% (1)
tr|A0A1S3EUN6|A0A1S3EUN6_DIPOR tRNA-dihydrouridine(20) synthase [NAD(P)+]-like OS=Dipodomys ordii OX=10020 GN=Dus2 PE=4 SV=1	tr|A0A1S3EUN6|A0A1S3EUN6_DIPOR	Dus2	43 kDa	99% (1)
tr|A0A1S3GID6|A0A1S3GID6_DIPOR S-adenosyl-L-methionine-dependent tRNA 4-demethylwyosine synthase-like OS=Dipodomys ordii OX=10020 GN=LOC105998465 PE=4 SV=1	tr|A0A1S3GID6|A0A1S3GID6_DIPOR	LOC105998465	80 kDa	99% (1)
tr|F1M8A9|F1M8A9_RAT Peptidyl-tRNA hydrolase 1 homolog OS=Rattus norvegicus OX=10116 GN=Ptrh1 PE=4 SV=1	tr|F1M8A9|F1M8A9_RAT	Ptrh1	22 kDa	99% (1)
tr|G5AJX3|G5AJX3_HETGA Lysine--tRNA ligase OS=Heterocephalus glaber OX=10181 GN=GW7_15127 PE=3 SV=1	tr|G5AJX3|G5AJX3_HETGA	GW7_15127	63 kDa	99% (1)
tr|O35880|O35880_RAT Isoleucyl tRNA synthetase OS=Rattus norvegicus OX=10116 PE=2 SV=1	tr|O35880|O35880_RAT		6 kDa	99% (1)

RNA polymerase:

tr|A0A091E2E8|A0A091E2E8_FUKDA RNA polymerase I-specific transcription initiation factor RRN3 OS=Fukomys damarensis OX=885580 GN=H920_01707 PE=4 SV=1	tr|A0A091E2E8|A0A091E2E8_FUKDA	H920_01707	79 kDa	99% (1)
tr|A0A1S3FGA8|A0A1S3FGA8_DIPOR DNA-directed RNA polymerase subunit beta OS=Dipodomys ordii OX=10020 GN=Polr1b PE=3 SV=1	tr|A0A1S3FGA8|A0A1S3FGA8_DIPOR	Polr1b	128 kDa	99% (1)
tr|A0A1S3G4R2|A0A1S3G4R2_DIPOR mediator of RNA polymerase II transcription subunit 26 isoform X1 OS=Dipodomys ordii OX=10020 GN=Med26 PE=4 SV=1	tr|A0A1S3G4R2|A0A1S3G4R2_DIPOR (+1)	Med26	63 kDa	99% (1)
tr|A0A1S3GI13|A0A1S3GI13_DIPOR DNA-directed DNA/RNA polymerase mu OS=Dipodomys ordii OX=10020 GN=Polm PE=3 SV=1	tr|A0A1S3GI13|A0A1S3GI13_DIPOR	Polm	55 kDa	99% (1)
tr|A0A1S3GIR3|A0A1S3GIR3_DIPOR DNA-directed RNA polymerase III subunit RPC9 OS=Dipodomys ordii OX=10020 GN=Crcp PE=4 SV=1	tr|A0A1S3GIR3|A0A1S3GIR3_DIPOR	Crcp	17 kDa	99% (1)
tr|G5C0Z7|G5C0Z7_HETGA Mediator of RNA polymerase II transcription subunit 12-like protein OS=Heterocephalus glaber OX=10181 GN=GW7_20769 PE=4 SV=1	tr|G5C0Z7|G5C0Z7_HETGA	GW7_20769	98 kDa	99% (1)
tr|G5C9B1|G5C9B1_HETGA TAF5-like RNA polymerase II p300/CBP-associated factor-associated factor 65 kDa subunit 5L OS=Heterocephalus glaber OX=10181 GN=GW7_06489 PE=4 SV=1	tr|G5C9B1|G5C9B1_HETGA	GW7_06489	40 kDa	99% (1)

Heat shock proteins:

sp|Q9QZ58|HSPB3_RAT Heat shock protein beta-3 OS=Rattus norvegicus OX=10116 GN=Hspb3 PE=2 SV=1	sp|Q9QZ58|HSPB3_RAT	Hspb3	17 kDa	99% (1)
tr|A0A0G2JVE2|A0A0G2JVE2_RAT DnaJ heat shock protein family (Hsp40) member C15 OS=Rattus norvegicus OX=10116 GN=Dnajc15 PE=1 SV=1	tr|A0A0G2JVE2|A0A0G2JVE2_RAT (+1)	Dnajc15	15 kDa	99% (1)
tr|A0A1S3EUY4|A0A1S3EUY4_DIPOR heat shock factor protein 4 isoform X3 OS=Dipodomys ordii OX=10020 GN=Hsf4 PE=3 SV=1	tr|A0A1S3EUY4|A0A1S3EUY4_DIPOR (+4)	Hsf4	49 kDa	99% (1)
tr|A0A1S3GEJ4|A0A1S3GEJ4_DIPOR heat shock 70 kDa protein 4 OS=Dipodomys ordii OX=10020 GN=Hspa4 PE=3 SV=1	tr|A0A1S3GEJ4|A0A1S3GEJ4_DIPOR	Hspa4	60 kDa	99% (1)
tr|A0A1S3GP00|A0A1S3GP00_DIPOR heat shock 70 kDa protein 1B OS=Dipodomys ordii OX=10020 GN=LOC105999841 PE=3 SV=1	tr|A0A1S3GP00|A0A1S3GP00_DIPOR	LOC105999841	70 kDa	99% (1)
tr|G5ARG8|G5ARG8_HETGA Heat shock 70 kDa protein 12A OS=Heterocephalus glaber OX=10181 GN=GW7_21295 PE=4 SV=1	tr|G5ARG8|G5ARG8_HETGA	GW7_21295	77 kDa	99% (1)

Translation initiation:

tr|A0A1S3FWP2|A0A1S3FWP2_DIPOR Eukaryotic translation initiation factor 3 subunit L OS=Dipodomys ordii OX=10020 GN=Eif3l PE=3 SV=1	tr|A0A1S3FWP2|A0A1S3FWP2_DIPOR	Eif3l	61 kDa	99% (1)
tr|A0A1S3GRE5|A0A1S3GRE5_DIPOR LOW QUALITY PROTEIN: eukaryotic translation initiation factor 4E transporter-like OS=Dipodomys ordii OX=10020 GN=LOC105999910 PE=4 SV=1	tr|A0A1S3GRE5|A0A1S3GRE5_DIPOR	LOC105999910	104 kDa	99% (1)
tr|G5C4X2|G5C4X2_HETGA Eukaryotic translation initiation factor 4 gamma 3 OS=Heterocephalus glaber OX=10181 GN=GW7_01849 PE=4 SV=1	tr|G5C4X2|G5C4X2_HETGA	GW7_01849	177 kDa	99% (1)

Calcium

tr|A0A1S3EP39|A0A1S3EP39_DIPOR EF-hand calcium-binding domain-containing protein 8 OS=Dipodomys ordii OX=10020 GN=Efcab8 PE=4 SV=1	tr|A0A1S3EP39|A0A1S3EP39_DIPOR	Efcab8	118 kDa	99% (2)
sp|P27732-10|CAC1D_RAT Isoform 10 of Voltage-dependent L-type calcium channel subunit alpha-1D OS=Rattus norvegicus OX=10116 GN=Cacna1d	sp|P27732-10|CAC1D_RAT (+19)	Cacna1d	245 kDa	99% (2)
sp|Q66H96|CABP7_RAT Calcium-binding protein 7 OS=Rattus norvegicus OX=10116 GN=Cabp7 PE=1 SV=1	sp|Q66H96|CABP7_RAT (+2)	Cabp7	24 kDa	99% (1)
sp|Q68FX6|CABS1_RAT Calcium-binding and spermatid-specific protein 1 OS=Rattus norvegicus OX=10116 GN=Cabs1 PE=1 SV=1	sp|Q68FX6|CABS1_RAT	Cabs1	42 kDa	99% (1)
sp|Q9WTN5|TPC1_RAT Two pore calcium channel protein 1 OS=Rattus norvegicus OX=10116 GN=Tpcn1 PE=1 SV=2	sp|Q9WTN5|TPC1_RAT	Tpcn1	94 kDa	99% (1)
tr|A0A091DDJ5|A0A091DDJ5_FUKDA Calcium-binding protein 2 OS=Fukomys damarensis OX=885580 GN=H920_10191 PE=4 SV=1	tr|A0A091DDJ5|A0A091DDJ5_FUKDA (+1)	H920_10191	28 kDa	99% (1)
tr|A0A091DKA1|A0A091DKA1_FUKDA EF-hand calcium-binding domain-containing protein 7 OS=Fukomys damarensis OX=885580 GN=H920_15351 PE=4 SV=1	tr|A0A091DKA1|A0A091DKA1_FUKDA	H920_15351	74 kDa	99% (1)
tr|A0A091E0N8|A0A091E0N8_FUKDA Calcium-dependent secretion activator 2 OS=Fukomys damarensis OX=885580 GN=H920_09976 PE=4 SV=1	tr|A0A091E0N8|A0A091E0N8_FUKDA	H920_09976	24 kDa	99% (1)
tr|A0A1S3ENV9|A0A1S3ENV9_DIPOR sodium/potassium/calcium exchanger 5 OS=Dipodomys ordii OX=10020 GN=Slc24a5 PE=3 SV=1	tr|A0A1S3ENV9|A0A1S3ENV9_DIPOR	Slc24a5	55 kDa	99% (1)
tr|A0A1S3ESA2|A0A1S3ESA2_DIPOR calcium-regulated heat stable protein 1 OS=Dipodomys ordii OX=10020 GN=Carhsp1 PE=4 SV=1	tr|A0A1S3ESA2|A0A1S3ESA2_DIPOR	Carhsp1	16 kDa	99% (1)
tr|A0A1S3F932|A0A1S3F932_DIPOR calcium homeostasis modulator protein 3 OS=Dipodomys ordii OX=10020 GN=Calhm3 PE=4 SV=1	tr|A0A1S3F932|A0A1S3F932_DIPOR	Calhm3	39 kDa	99% (1)
tr|A0A1S3FSE8|A0A1S3FSE8_DIPOR calcium-independent phospholipase A2-gamma isoform X1 OS=Dipodomys ordii OX=10020 GN=Pnpla8 PE=4 SV=1	tr|A0A1S3FSE8|A0A1S3FSE8_DIPOR (+2)	Pnpla8	88 kDa	99% (1)
tr|A0A1S3FTA4|A0A1S3FTA4_DIPOR voltage-dependent calcium channel subunit alpha-2/delta-4 OS=Dipodomys ordii OX=10020 GN=Cacna2d4 PE=4 SV=1	tr|A0A1S3FTA4|A0A1S3FTA4_DIPOR	Cacna2d4	119 kDa	99% (1)
tr|A0A1S3GWA7|A0A1S3GWA7_DIPOR calcium and integrin-binding protein 1 OS=Dipodomys ordii OX=10020 GN=Cib1 PE=4 SV=1	tr|A0A1S3GWA7|A0A1S3GWA7_DIPOR	Cib1	23 kDa	99% (1)
