# Supplementary material for: Activation of Gαq sequesters specific transcripts into Ago2 particles
Source: Sci Rep. 2022 May 24;12:8758. doi: 10.1038/s41598-022-12737-w (PMC9130320; doi:10.1038/s41598-022-12737-w)
Supplement: Supplementary file 6 — Supplementary Information 6. [file 41598_2022_12737_MOESM6_ESM.pdf]

SI Table 5: mRNA bound to Ago2 in carbachol stimulated cells

					peak_info					
	Chromosome	Start	End	-log10(P-value)	Log2 Fold Change	Strand	Gene	Ensembl ID	Feature	Size
1	chr3	125440048	125440054	400	7.315979903	+	Chgb	ENSRNOG00000021269	CDS	6
2	chr3	125440002	125440022	400	7.293964101	+	Chgb	ENSRNOG00000021269	CDS	20
3	chr3	125440042	125440048	400	7.273717867	+	Chgb	ENSRNOG00000021269	CDS	6
4	chr3	125440028	125440038	400	7.090682783	+	Chgb	ENSRNOG00000021269	CDS	10
5	chr3	125440038	125440042	400	7.08000105	+	Chgb	ENSRNOG00000021269	CDS	4
6	chr3	125440022	125440028	400	7.010683461	+	Chgb	ENSRNOG00000021269	CDS	6
7	chr6	126443051	126443062	318.4022956	8.777086442	+	Chga	ENSRNOG00000052549	CDS	11
8	chr7	2505371	2505388	311.1491389	9.73302165	+	Atp5f1b	ENSRNOG00000002840	CDS	17
9	chr7	2505388	2505408	310.3494301	8.741307529	+	Atp5f1b	ENSRNOG00000002840	CDS	20
10	chr6	126443062	126443080	298.4888748	8.686248511	+	Chga	ENSRNOG00000052549	CDS	18
11	chr6	126443015	126443044	293.8682104	8.663622756	+	Chga	ENSRNOG00000052549	CDS	29
12	chr7	2505408	2505423	290.2766379	8.646078072	+	Atp5f1b	ENSRNOG00000002840	CDS	15
13	chr7	2505423	2505436	283.0452603	8.611031125	+	Atp5f1b	ENSRNOG00000002840	CDS	13
14	chr6	126443080	126443105	281.5995358	8.602659564	+	Chga	ENSRNOG00000052549	CDS	25
15	chr7	2505353	2505371	281.4760203	9.590716074	+	Atp5f1b	ENSRNOG00000002840	CDS	18
16	chr3	125439973	125439994	276.2037601	7.598455496	+	Chgb	ENSRNOG00000021269	CDS	21
17	chr6	126443044	126443051	273.3652518	8.560777652	+	Chga	ENSRNOG00000052549	CDS	7
18	chr3	125440057	125440081	264.6138449	7.538274783	+	Chgb	ENSRNOG00000021269	CDS	24
19	chr3	125439994	125439998	262.4969533	7.526521601	+	Chgb	ENSRNOG00000021269	CDS	4
20	chr3	125439050	125439095	244.4937602	9.388870555	+	Chgb	ENSRNOG00000021269	CDS	45
21	chr3	125439138	125439151	240.898144	9.367618006	+	Chgb	ENSRNOG00000021269	CDS	13
22	chr3	125439124	125439138	239.6296228	9.360187801	+	Chgb	ENSRNOG00000021269	CDS	14
23	chr3	125439998	125440002	238.9017312	7.395347312	+	Chgb	ENSRNOG00000021269	CDS	4
24	chr3	125440054	125440057	237.3955251	7.387246813	+	Chgb	ENSRNOG00000021269	CDS	3
25	chr3	125439095	125439110	234.9654518	9.331767628	+	Chgb	ENSRNOG00000021269	CDS	15
26	chr3	125439110	125439116	233.0722694	9.320751401	+	Chgb	ENSRNOG00000021269	CDS	6
27	chr7	2505436	2505440	229.4113409	8.313075205	+	Atp5f1b	ENSRNOG00000002840	CDS	4
28	chr7	2505339	2505353	225.6635385	9.274947711	+	Atp5f1b	ENSRNOG00000002840	CDS	14
29	chr3	125439120	125439124	223.7701201	9.262601835	+	Chgb	ENSRNOG00000021269	CDS	4
30	chr3	125439116	125439120	221.2261907	9.246571725	+	Chgb	ENSRNOG00000021269	CDS	4
31	chr3	125439252	125439288	221.1514713	8.261715931	+	Chgb	ENSRNOG00000021269	CDS	36
32	chr3	125439151	125439156	211.7197028	9.183417996	+	Chgb	ENSRNOG00000021269	CDS	5
33	chr3	125439195	125439218	206.3950491	9.148378718	+	Chgb	ENSRNOG00000021269	CDS	23
34	chr3	125439156	125439159	205.1132045	9.139725406	+	Chgb	ENSRNOG00000021269	CDS	3
35	chr3	125439245	125439252	203.4326928	8.143577732	+	Chgb	ENSRNOG00000021269	CDS	7
36	chr3	125440081	125440087	203.3436704	7.572017936	+	Chgb	ENSRNOG00000021269	CDS	6
37	chr3	125439962	125439973	202.0782953	7.563416217	+	Chgb	ENSRNOG00000021269	CDS	11
38	chr7	2505440	2505467	194.5750207	8.08067098	+	Atp5f1b	ENSRNOG00000002840	CDS	27
39	chr3	125439159	125439165	193.5217424	9.05635532	+	Chgb	ENSRNOG00000021269	CDS	6
40	chr6	126443105	126443119	192.4478478	8.065521786	+	Chga	ENSRNOG00000052549	CDS	14
41	chr3	125440087	125440098	189.8477609	7.476492489	+	Chgb	ENSRNOG00000021269	CDS	11
42	chr7	117240548	117240575	189.6491409	7.474454783	-	Plec	ENSRNOG00000023781	CDS	27
43	chr7	2505294	2505339	187.6260089	9.011737678	+	Atp5f1b	ENSRNOG00000002840	CDS	45
44	chr6	126442979	126443015	187.4144896	8.027459174	+	Chga	ENSRNOG00000052549	CDS	36
45	chr3	125440466	125440508	184.6774342	8.006458875	+	Chgb	ENSRNOG00000021269	CDS	42
46	chr3	125440098	125440109	181.0925624	7.978693347	+	Chgb	ENSRNOG00000021269	CDS	11
47	chr3	125439653	125439664	180.8667492	7.977614697	+	Chgb	ENSRNOG00000021269	CDS	11
48	chr7	117240532	117240538	180.670628	7.975454973	-	Plec	ENSRNOG00000023781	CDS	6
49	chr3	125439241	125439245	180.0174697	7.971125802	+	Chgb	ENSRNOG00000021269	CDS	4
50	chr7	117240538	117240548	179.7325643	7.400185869	-	Plec	ENSRNOG00000023781	CDS	10

SI Table 5: mRNA bound to Ago2 in cells receiving heat shock

<table>
<tr><th></th><th></th><th></th><th></th><th></th><th></th><th>peak_info</th><th></th><th></th><th></th><th></th></tr>
<tr><th></th><th>Chromosome</th><th>Start</th><th>End</th><th>-log10(P-value)</th><th>Log2 Fold Change</th><th>Strand</th><th>Gene</th><th>Ensembl ID</th><th>Feature</th><th>Size</th></tr>
<tr><td>1</td><td>chr12</td><td>13717998</td><td>13718070</td><td>400</td><td>4.068446687</td><td>+</td><td>Actb</td><td>ENSRNOG00000034254</td><td>CDS</td><td>72</td></tr>
<tr><td>2</td><td>chr12</td><td>13717957</td><td>13717998</td><td>400</td><td>4.047667459</td><td>+</td><td>Actb</td><td>ENSRNOG00000034254</td><td>CDS</td><td>41</td></tr>
<tr><td>3</td><td>chr20</td><td>17075852</td><td>17075995</td><td>400</td><td>4.421478252</td><td>-</td><td>Zwint</td><td>ENSRNOG00000048682</td><td>3' UTR</td><td>143</td></tr>
<tr><td>4</td><td>chr7</td><td>121302265</td><td>121302299</td><td>400</td><td>4.705271218</td><td>-</td><td>RF00221|AC127784.1</td><td>ENSRNOG00000052611||ENSRNOG00000059190</td><td>Proximal intron</td><td>34</td></tr>
<tr><td>5</td><td>chr7</td><td>121302237</td><td>121302265</td><td>400</td><td>4.705271218</td><td>-</td><td>AC127784.1|RF00221</td><td>ENSRNOG00000052611||ENSRNOG00000059190</td><td>Noncoding exon</td><td>28</td></tr>
<tr><td>6</td><td>chrM</td><td>7389</td><td>7572</td><td>400</td><td>4.304729443</td><td>+</td><td>COX2</td><td>ENSRNOG00000030371</td><td>CDS</td><td>183</td></tr>
<tr><td>7</td><td>chrM</td><td>7572</td><td>7601</td><td>400</td><td>4.158519398</td><td>+</td><td>COX2</td><td>ENSRNOG00000030371</td><td>CDS</td><td>29</td></tr>
<tr><td>8</td><td>chrM</td><td>5113</td><td>5141</td><td>400</td><td>6.176040806</td><td>-</td><td>AY172581.18</td><td>ENSRNOG00000032609</td><td>Noncoding exon</td><td>28</td></tr>
<tr><td>9</td><td>chrM</td><td>3760</td><td>3766</td><td>400</td><td>4.952820914</td><td>-</td><td>chrM.trna4-GlnTTG</td><td>chrM.trna4-GlnTTG</td><td>tRNA</td><td>6</td></tr>
<tr><td>10</td><td>chrM</td><td>3789</td><td>3816</td><td>400</td><td>4.704893401</td><td>-</td><td>chrM.trna4-GlnTTG</td><td>chrM.trna4-GlnTTG</td><td>tRNA</td><td>27</td></tr>
<tr><td>11</td><td>chr9</td><td>82161735</td><td>82161746</td><td>255.7574996</td><td>10.32597433</td><td>-</td><td>rno-mir-375</td><td>MI0006140</td><td>miRNA</td><td>11</td></tr>
<tr><td>12</td><td>chr9</td><td>82161726</td><td>82161735</td><td>255.7574996</td><td>10.32597433</td><td>-</td><td>rno-mir-375</td><td>MI0006140</td><td>miRNA</td><td>9</td></tr>
<tr><td>13</td><td>chr9</td><td>82161724</td><td>82161726</td><td>245.5614482</td><td>10.26777945</td><td>-</td><td>rno-mir-375</td><td>MI0006140</td><td>miRNA</td><td>2</td></tr>
<tr><td>14</td><td>chr17</td><td>823527</td><td>823535</td><td>181.6484802</td><td>7.881562231</td><td>+</td><td>rno-mir-27b</td><td>MI0000859</td><td>miRNA</td><td>8</td></tr>
<tr><td>15</td><td>chr17</td><td>823535</td><td>823542</td><td>181.6484802</td><td>7.881562231</td><td>+</td><td>rno-mir-27b</td><td>MI0000859</td><td>miRNA</td><td>7</td></tr>
<tr><td>16</td><td>chr17</td><td>823520</td><td>823527</td><td>181.6484802</td><td>7.881562231</td><td>+</td><td>rno-mir-27b</td><td>MI0000859</td><td>miRNA</td><td>7</td></tr>
<tr><td>17</td><td>chr19</td><td>25318797</td><td>25318805</td><td>145.4657355</td><td>9.520295988</td><td>+</td><td>rno-mir-27a</td><td>MI0000860</td><td>miRNA</td><td>8</td></tr>
<tr><td>18</td><td>chr19</td><td>25318805</td><td>25318812</td><td>145.4657355</td><td>9.520295988</td><td>+</td><td>rno-mir-27a</td><td>MI0000860</td><td>miRNA</td><td>7</td></tr>
<tr><td>19</td><td>chr19</td><td>25318790</td><td>25318797</td><td>145.4657355</td><td>9.520295988</td><td>+</td><td>rno-mir-27a</td><td>MI0000860</td><td>miRNA</td><td>7</td></tr>
<tr><td>20</td><td>chr2</td><td>188405733</td><td>188405782</td><td>79.0504246</td><td>4.321006067</td><td>-</td><td>Fdps</td><td>ENSRNOG00000043377</td><td>CDS</td><td>49</td></tr>
<tr><td>21</td><td>chr17</td><td>42584117</td><td>42584261</td><td>76.05749589</td><td>5.970098906</td><td>-</td><td>Cmahp</td><td>ENSRNOG00000003094</td><td>3' UTR</td><td>144</td></tr>
<tr><td>22</td><td>chr8</td><td>52189402</td><td>52189451</td><td>74.26085504</td><td>4.758353728</td><td>+</td><td>Cadm1</td><td>ENSRNOG00000018778</td><td>3' UTR</td><td>49</td></tr>
<tr><td>23</td><td>chr8</td><td>52189451</td><td>52189509</td><td>68.42428116</td><td>5.162743984</td><td>+</td><td>Cadm1</td><td>ENSRNOG00000018778</td><td>3' UTR</td><td>58</td></tr>
<tr><td>24</td><td>chr17</td><td>823288</td><td>823301</td><td>67.84578546</td><td>7.472599246</td><td>+</td><td>rno-mir-23b</td><td>MI0000853</td><td>miRNA</td><td>13</td></tr>
<tr><td>25</td><td>chr17</td><td>823277</td><td>823288</td><td>66.36077676</td><td>6.887636745</td><td>+</td><td>rno-mir-23b</td><td>MI0000853</td><td>miRNA</td><td>11</td></tr>
<tr><td>26</td><td>chr20</td><td>7191420</td><td>7191584</td><td>66.16995194</td><td>6.49664472</td><td>-</td><td>Nudt3</td><td>ENSRNOG00000061176</td><td>Distal intron</td><td>164</td></tr>
<tr><td>27</td><td>chr20</td><td>17076296</td><td>17076401</td><td>61.99046412</td><td>3.445677947</td><td>-</td><td>Zwint</td><td>ENSRNOG00000048682</td><td>3' UTR</td><td>105</td></tr>
<tr><td>28</td><td>chr20</td><td>17075995</td><td>17076101</td><td>61.78099212</td><td>3.259605523</td><td>-</td><td>Zwint</td><td>ENSRNOG00000048682</td><td>3' UTR</td><td>106</td></tr>
<tr><td>29</td><td>chr2</td><td>188405682</td><td>188405733</td><td>59.16032088</td><td>4.037213101</td><td>-</td><td>Fdps</td><td>ENSRNOG00000043377</td><td>CDS</td><td>51</td></tr>
<tr><td>30</td><td>chr2</td><td>188405822</td><td>188405886</td><td>58.86626953</td><td>3.191890329</td><td>-</td><td>Fdps</td><td>ENSRNOG00000043377</td><td>CDS</td><td>64</td></tr>
<tr><td>31</td><td>chr16</td><td>20430444</td><td>20430563</td><td>53.4097716</td><td>6.592731824</td><td>+</td><td>Ifi30</td><td>ENSRNOG00000019387</td><td>3' UTR</td><td>119</td></tr>
<tr><td>32</td><td>chr2</td><td>188405782</td><td>188405822</td><td>53.10797779</td><td>3.6932587</td><td>-</td><td>Fdps</td><td>ENSRNOG00000043377</td><td>CDS</td><td>40</td></tr>
<tr><td>33</td><td>chr3</td><td>161302411</td><td>161302510</td><td>47.07778333</td><td>4.577781483</td><td>+</td><td>Ctsa</td><td>ENSRNOG00000015857</td><td>CDS</td><td>99</td></tr>
<tr><td>34</td><td>chr11</td><td>81378404</td><td>81378448</td><td>46.70843137</td><td>4.369194861</td><td>-</td><td>RF01299</td><td>ENSRNOG00000053621</td><td>Proximal intron</td><td>44</td></tr>
<tr><td>35</td><td>chr11</td><td>81378398</td><td>81378404</td><td>46.07719153</td><td>4.353075196</td><td>-</td><td>RF01299</td><td>ENSRNOG00000053621</td><td>Noncoding exon</td><td>6</td></tr>
<tr><td>36</td><td>chr1</td><td>22758916</td><td>22758954</td><td>43.5559552</td><td>4.286732701</td><td>+</td><td>RF00186</td><td>ENSRNOG00000057952</td><td>Proximal intron</td><td>38</td></tr>
<tr><td>37</td><td>chr1</td><td>22758954</td><td>22758981</td><td>42.77448396</td><td>4.179817497</td><td>+</td><td>RF00186</td><td>ENSRNOG00000057952</td><td>Noncoding exon</td><td>27</td></tr>
<tr><td>38</td><td>chr7</td><td>140637383</td><td>140637520</td><td>42.61305538</td><td>3.724622871</td><td>-</td><td>Tuba1a</td><td>ENSRNOG00000060728</td><td>CDS</td><td>137</td></tr>
<tr><td>39</td><td>chr1</td><td>22758981</td><td>22758987</td><td>41.52196768</td><td>4.145465992</td><td>+</td><td>RF00186</td><td>ENSRNOG00000057952</td><td>Noncoding exon</td><td>6</td></tr>
<tr><td>40</td><td>chr11</td><td>81378448</td><td>81378463</td><td>41.52196768</td><td>4.145465992</td><td>-</td><td>RF01299</td><td>ENSRNOG00000053621</td><td>Noncoding exon</td><td>15</td></tr>
<tr><td>41</td><td>chr17</td><td>16420045</td><td>16420056</td><td>41.49396632</td><td>7.747706484</td><td>+</td><td>rno-let-7d</td><td>MI0000601</td><td>miRNA</td><td>11</td></tr>
<tr><td>42</td><td>chr16</td><td>20430382</td><td>20430444</td><td>40.5095868</td><td>5.844568023</td><td>+</td><td>Ifi30</td><td>ENSRNOG00000019387</td><td>3' UTR</td><td>62</td></tr>
<tr><td>43</td><td>chr17</td><td>16420056</td><td>16420068</td><td>40.04922758</td><td>6.747706484</td><td>+</td><td>rno-let-7d</td><td>MI0000601</td><td>miRNA</td><td>12</td></tr>
<tr><td>44</td><td>chr9</td><td>16621062</td><td>16621294</td><td>38.87208182</td><td>6.7070645</td><td>+</td><td>Rrp36</td><td>ENSRNOG00000017836</td><td>CDS</td><td>232</td></tr>
<tr><td>45</td><td>chr3</td><td>2519380</td><td>2519416</td><td>37.334057</td><td>7.600149296</td><td>-</td><td>Grin1</td><td>ENSRNOG00000011726</td><td>5' splice site</td><td>36</td></tr>
<tr><td>46</td><td>chr2</td><td>165606096</td><td>165606111</td><td>35.55267805</td><td>7.531977793</td><td>+</td><td>rno-mir-16</td><td>MI0000844</td><td>miRNA</td><td>15</td></tr>
<tr><td>47</td><td>chr2</td><td>165606085</td><td>165606096</td><td>35.55267805</td><td>7.531977793</td><td>+</td><td>rno-mir-16</td><td>MI0000844</td><td>miRNA</td><td>11</td></tr>
<tr><td>48</td><td>chr3</td><td>2519416</td><td>2519463</td><td>33.58656125</td><td>5.600149296</td><td>-</td><td>Grin1</td><td>ENSRNOG00000011726</td><td>CDS</td><td>47</td></tr>
<tr><td>49</td><td>chr14</td><td>114167236</td><td>114167258</td><td>32.40624887</td><td>4.50851882</td><td>+</td><td>Rtn4</td><td>ENSRNOG00000004621</td><td>CDS</td><td>22</td></tr>
<tr><td>50</td><td>chr2</td><td>2635163</td><td>2635296</td><td>31.239074</td><td>6.410671497</td><td>-</td><td>Rhobtb3</td><td>ENSRNOG00000012414</td><td>CDS</td><td>133</td></tr>
</table>
