# Supplementary material for: Activation of Gαq sequesters specific transcripts into Ago2 particles
Source: Sci Rep. 2022 May 24;12:8758. doi: 10.1038/s41598-022-12737-w (PMC9130320; doi:10.1038/s41598-022-12737-w)
Supplement: Supplementary file 7 — Supplementary Information 7. [file 41598_2022_12737_MOESM7_ESM.pdf]

SI Table 6 Ago2 bound RNAs from cells subjected to heat shock

	Chromosome	Start	End	-log10(P-value)	Log2 Fold Change	Strand	Gene	Ensembl ID	Feature	Size
1	chr12	13717998	13718070	400	4.068446687	+	Actb	ENSRNOG00000034254	CDS	72
2	chr12	13717957	13717998	400	4.047667459	+	Actb	ENSRNOG00000034254	CDS	41
3	chr20	17075852	17075995	400	4.421478252	-	Zwint	ENSRNOG00000048682	3' UTR	143
4	chr7	121302265	121302299	400	4.705271218	-	RF00221|AC127784.1	ENSRNOG00000052611||ENSRNOG00000059190	Proximal intron	34
5	chr7	121302237	121302265	400	4.705271218	-	AC127784.1|RF00221	ENSRNOG00000052611||ENSRNOG00000059190	Noncoding exon	28
6	chrM	7389	7572	400	4.304729443	+	COX2	ENSRNOG00000030371	CDS	183
7	chrM	7572	7601	400	4.158519398	+	COX2	ENSRNOG00000030371	CDS	29
8	chrM	5113	5141	400	6.176040806	-	AY172581.18	ENSRNOG00000032609	Noncoding exon	28
9	chrM	3760	3766	400	4.952820914	-	chrM.trna4-GlnTTG	chrM.trna4-GlnTTG	tRNA	6
10	chrM	3789	3816	400	4.704893401	-	chrM.trna4-GlnTTG	chrM.trna4-GlnTTG	tRNA	27
11	chr9	82161735	82161746	255.7574996	10.32597433	-	rno-mir-375	MI0006140	miRNA	11
12	chr9	82161726	82161735	255.7574996	10.32597433	-	rno-mir-375	MI0006140	miRNA	9
13	chr9	82161724	82161726	245.5614482	10.26777945	-	rno-mir-375	MI0006140	miRNA	2
14	chr17	823527	823535	181.6484802	7.881562231	+	rno-mir-27b	MI0000859	miRNA	8
15	chr17	823535	823542	181.6484802	7.881562231	+	rno-mir-27b	MI0000859	miRNA	7
16	chr17	823520	823527	181.6484802	7.881562231	+	rno-mir-27b	MI0000859	miRNA	7
17	chr19	25318797	25318805	145.4657355	9.520295988	+	rno-mir-27a	MI0000860	miRNA	8
18	chr19	25318805	25318812	145.4657355	9.520295988	+	rno-mir-27a	MI0000860	miRNA	7
19	chr19	25318790	25318797	145.4657355	9.520295988	+	rno-mir-27a	MI0000860	miRNA	7
20	chr2	188405733	188405782	79.0504246	4.321006067	-	Fdps	ENSRNOG00000043377	CDS	49
21	chr17	42584117	42584261	76.05749589	5.970098906	-	Cmahp	ENSRNOG00000003094	3' UTR	144
22	chr8	52189402	52189451	74.26085504	4.758353728	+	Cadm1	ENSRNOG00000018778	3' UTR	49
23	chr8	52189451	52189509	68.42428116	5.162743984	+	Cadm1	ENSRNOG00000018778	3' UTR	58
24	chr17	823288	823301	67.84578546	7.472599246	+	rno-mir-23b	MI0000853	miRNA	13
25	chr17	823277	823288	66.36077676	6.887636745	+	rno-mir-23b	MI0000853	miRNA	11
26	chr20	7191420	7191584	66.16995194	6.49664472	-	Nudt3	ENSRNOG00000061176	Distal intron	164
27	chr20	17076296	17076401	61.99046412	3.445677947	-	Zwint	ENSRNOG00000048682	3' UTR	105
28	chr20	17075995	17076101	61.78099212	3.259605523	-	Zwint	ENSRNOG00000048682	3' UTR	106
29	chr2	188405682	188405733	59.16032088	4.037213101	-	Fdps	ENSRNOG00000043377	CDS	51
30	chr2	188405822	188405886	58.86626953	3.191890329	-	Fdps	ENSRNOG00000043377	CDS	64
31	chr16	20430444	20430563	53.4097716	6.592731824	+	Ifi30	ENSRNOG00000019387	3' UTR	119
32	chr2	188405782	188405822	53.10797779	3.6932587	-	Fdps	ENSRNOG00000043377	CDS	40
33	chr3	161302411	161302510	47.07778333	4.577781483	+	Ctsa	ENSRNOG00000015857	CDS	99
34	chr11	81378404	81378448	46.70843137	4.369194861	-	RF01299	ENSRNOG00000053621	Proximal intron	44
35	chr11	81378398	81378404	46.07719153	4.353075196	-	RF01299	ENSRNOG00000053621	Noncoding exon	6
36	chr1	22758916	22758954	43.5559552	4.286732701	+	RF00186	ENSRNOG00000057952	Proximal intron	38
37	chr1	22758954	22758981	42.77448396	4.179817497	+	RF00186	ENSRNOG00000057952	Noncoding exon	27
38	chr7	140637383	140637520	42.61305538	3.724622871	-	Tuba1a	ENSRNOG00000060728	CDS	137
39	chr1	22758981	22758987	41.52196768	4.145465992	+	RF00186	ENSRNOG00000057952	Noncoding exon	6
40	chr11	81378448	81378463	41.52196768	4.145465992	-	RF01299	ENSRNOG00000053621	Noncoding exon	15
41	chr17	16420045	16420056	41.49396632	7.747706484	+	rno-let-7d	MI0000601	miRNA	11
42	chr16	20430382	20430444	40.5095868	5.844568023	+	Ifi30	ENSRNOG00000019387	3' UTR	62
43	chr17	16420056	16420068	40.04922758	6.747706484	+	rno-let-7d	MI0000601	miRNA	12
44	chr9	16621062	16621294	38.87208182	6.7070645	+	Rrp36	ENSRNOG00000017836	CDS	232
45	chr3	2519380	2519416	37.334057	7.600149296	-	Grin1	ENSRNOG00000011726	5' splice site	36
46	chr2	165606096	165606111	35.55267805	7.531977793	+	rno-mir-16	MI0000844	miRNA	15

47	chr2	165606085	165606096	35.55267805	7.531977793	+	rno-mir-16	MI0000844	miRNA	11
48	chr3	2519416	2519463	33.58656125	5.600149296	-	Grin1	ENSRNOG00000011726	CDS	47
49	chr14	114167236	114167258	32.40624887	4.50851882	+	Rtn4	ENSRNOG00000004621	CDS	22
50	chr2	2635163	2635296	31.239074	6.410671497	-	Rhobtb3	ENSRNOG00000012414	CDS	133
51	chrM	15360	15380	31.19741782	3.445677947	-	AY172581.2	ENSRNOG00000029145	Noncoding exon	20
52	chrM	15350	15360	31.19741782	3.445677947	-	AY172581.2	ENSRNOG00000029145	Noncoding exon	10
53	chr19	25318636	25318649	30.21485425	7.305701937	+	rno-mir-23a	MI0000852	miRNA	13
54	chr19	25318626	25318636	30.21485425	7.305701937	+	rno-mir-23a	MI0000852	miRNA	10
55	chr1	101449433	101449439	30.00703138	3.405600507	-	Ftl1	ENSRNOG00000020843	CDS	6
56	chr12	39871263	39871371	29.23674426	4.385136405	-	Ppp1cc	ENSRNOG00000001269	CDS	108
57	chr1	197986800	197986899	28.97819072	4.720739437	-	Cln3	ENSRNOG00000019103	CDS	99
58	chr3	161302360	161302411	28.93479387	4.113834383	+	Ctsa	ENSRNOG00000015857	CDS	51
59	chr1	228364699	228364831	27.91578131	4.899709578	+	Patl1	ENSRNOG00000021052	CDS	132
60	chr2	27481243	27481394	27.78012744	3.555061406	-	Hmgcr	ENSRNOG00000016122	3' UTR	151
61	chr2	123162531	123162674	27.68526927	4.06320831	-	Anxa5	ENSRNOG00000014453	3' UTR	143
62	chr11	24425123	24425185	27.27417001	4.870563232	-	App	ENSRNOG00000006997	3' UTR	62
63	chr8	52189311	52189402	27.07171171	3.352221782	+	Cadm1	ENSRNOG00000018778	3' UTR	91
64	chr20	17076479	17076532	26.48157819	3.329853969	-	Zwint	ENSRNOG00000048682	3' UTR	53
65	chr14	114167188	114167236	26.0807545	4.250206825	+	Rtn4	ENSRNOG00000004621	CDS	48
66	chr14	114167258	114167306	25.93979338	4.108296199	+	Rtn4	ENSRNOG00000004621	CDS	48
67	chr7	12243325	12243388	25.78773247	4.577781483	-	Reep6	ENSRNOG00000033262	CDS	63
68	chr14	115257453	115257571	25.60844743	4.385136405	+	Psme4	ENSRNOG00000060340	CDS	118
69	chr20	10674588	10674646	25.39604574	6.132370334	-	Sik1	ENSRNOG00000001189	CDS	58
70	chr8	22436344	22436422	24.81353697	6.101343439	+	Ilf3	ENSRNOG00000022741	Distal intron	78
71	chr10	20598590	20598666	24.51478981	4.516380938	+	Pank3	ENSRNOG00000007419	CDS	76
72	chr6	60771958	60772004	24.44608616	5.192491327	+	Dock4	ENSRNOG00000004823	CDS	46
73	chr2	27483137	27483238	23.86305759	3.790775206	-	Hmgcr	ENSRNOG00000016122	CDS	101
74	chr1	80920775	80920904	23.58169871	3.072546175	+	Zfp180	ENSRNOG00000029336	5' UTR	129
75	chr1	59704848	59704864	23.11749015	6.935333487	+	rno-mir-125a	MI0000895	miRNA	16
76	chr1	59704840	59704848	23.11749015	6.935333487	+	rno-mir-125a	MI0000895	miRNA	8
77	chr10	74025040	74025084	23.08172321	4.262279657	-	Cltc	ENSRNOG00000004291	CDS	44
78	chr1	101449727	101449747	23.06549145	3.498347015	-	Ftl1	ENSRNOG00000020843	CDS	20
79	chr19	55262320	55262410	22.72172667	3.840815889	-	Mvd	ENSRNOG00000013376	CDS	90
80	chr10	94982150	94982200	22.60961252	4.419083737	-	rno-mir-3064	MI0030357	miRNA proximal	50
81	chr19	37263212	37263235	22.48747596	5.970098906	-	rno-mir-328a	MI0000602	miRNA	23
82	chr9	10022547	10022637	22.18367642	5.069634579	+	Khsrp	ENSRNOG00000047628	Proximal intron	90
83	chr16	14373041	14373085	22.15473448	4.613405393	-	Ghitm	ENSRNOG00000013961	CDS	44
84	chr7	66802742	66802758	21.93756205	6.863183702	+	rno-let-7i	MI0000835	miRNA	16
85	chr7	66802734	66802742	21.93756205	6.863183702	+	rno-let-7i	MI0000835	miRNA	8
86	chr5	79373517	79373745	21.06943299	4.004046237	+	Atp6v1g1	ENSRNOG00000008163	3' UTR	228
87	chr9	10022382	10022480	20.87801362	3.747706484	+	Khsrp	ENSRNOG00000047628	Proximal intron	98
88	chr20	10674553	10674588	20.86267976	5.350370987	-	Sik1	ENSRNOG00000001189	CDS	35
89	chr6	60771910	60771958	20.86267976	5.350370987	+	Dock4	ENSRNOG00000004823	CDS	48
90	chr8	67396745	67396783	20.7587066	6.787234848	-	Coro2b	ENSRNOG00000015257	Distal intron	38
91	chr8	67396783	67396815	20.7587066	6.787234848	-	Coro2b	ENSRNOG00000015257	Distal intron	32
92	chr8	67396723	67396745	20.7587066	6.787234848	-	Coro2b	ENSRNOG00000015257	Distal intron	22
93	chr7	117240470	117240522	20.6784637	3.385136405	-	Plec	ENSRNOG00000023781	CDS	52

94	chr1	238847416	238847520	20.24299896	4.503780901	+	Zfand5	ENSRNOG00000018107	CDS	104
95	chrX	10559950	10559995	20.16868896	5.825708996	-	Usp9x	ENSRNOG00000003261	CDS	45
96	chr3	122703631	122703726	20.06497163	3.221637673	-	Snrpb	ENSRNOG00000006961	CDS	95
97	chr10	94982200	94982253	19.94100491	4.092354656	-	rno-mir-3064	MI0030357	Proximal intron	53
98	chr10	74025027	74025040	19.60702181	4.465306754	-	Cltc	ENSRNOG00000004291	CDS	13
99	chr8	22436422	22436436	19.59029879	5.787234848	+	Ilf3	ENSRNOG00000022741	Distal intron	14
100	chr18	15,225,173	15,225,215	19.58103799	6.7070645	-	Rnf125	ENSRNOG00000057832	CDS	42
